# Cigarette Smoke Extract Disturbs Mitochondria-Regulated Airway Epithelial Cell Responses to Pneumococci

**DOI:** 10.3390/cells11111771

**Published:** 2022-05-28

**Authors:** Mahyar Aghapour, Christy B. M. Tulen, Mohsen Abdi Sarabi, Sönke Weinert, Mathias Müsken, Borna Relja, Frederik-Jan van Schooten, Andreas Jeron, Rüdiger Braun-Dullaeus, Alexander H. Remels, Dunja Bruder

**Affiliations:** 1 Infection Immunology Group, Institute of Medical Microbiology, Infection Control and Prevention, Health Campus Immunology, Infectiology and Inflammation, Otto-von-Guericke University, 39120 Magdeburg, Germany; mahyar.aghapour@med.ovgu.de (M.A.); andreas.jeron@helmholtz-hzi.de (A.J.); 2 Immune Regulation Group, Helmholtz Centre for Infection Research, 38124 Braunschweig, Germany; 3 Department of Pharmacology and Toxicology, School of Nutrition and Translational Research in Metabolism (NUTRIM), Maastricht University Medical Center, 6229 ER Maastricht, The Netherlands; christy.tulen@maastrichtuniversity.nl (C.B.M.T.); f.vanschooten@maastrichtuniversity.nl (F.-J.v.S.); a.remels@maastrichtuniversity.nl (A.H.R.); 4 Department of Internal Medicine/Cardiology and Angiology, Otto-von-Guericke University, 39120 Magdeburg, Germany; mohsen.abdi@med.ovgu.de (M.A.S.); soenke.weinert@med.ovgu.de (S.W.); 5 Central Facility for Microscopy, Helmholtz Centre for Infection Research, 38124 Braunschweig, Germany; mathias.muesken@helmholtz-hzi.de; 6 Experimental Radiology, Department of Radiology and Nuclear Medicine, 39120 Magdeburg, Germany; borna.relja@med.ovgu.de

**Keywords:** mitochondrial dysfunction, cigarette smokes extract, lung epithelial cells, pneumococcal infection

## Abstract

Mitochondrial functionality is crucial for the execution of physiologic functions of metabolically active cells in the respiratory tract including airway epithelial cells (AECs). Cigarette smoke is known to impair mitochondrial function in AECs. However, the potential contribution of mitochondrial dysfunction in AECs to airway infection and airway epithelial barrier dysfunction is unknown. In this study, we used an in vitro model based on AECs exposed to cigarette smoke extract (CSE) followed by an infection with *Streptococcus pneumoniae* (*Sp*). The levels of oxidative stress as an indicator of mitochondrial stress were quantified upon CSE and *Sp* treatment. In addition, expression of proteins associated with mitophagy, mitochondrial content, and biogenesis as well as mitochondrial fission and fusion was quantified. Transcriptional AEC profiling was performed to identify the potential changes in innate immune pathways and correlate them with indices of mitochondrial function. We observed that CSE exposure substantially altered mitochondrial function in AECs by suppressing mitochondrial complex protein levels, reducing mitochondrial membrane potential and increasing mitochondrial stress and mitophagy. Moreover, CSE-induced mitochondrial dysfunction correlated with reduced enrichment of genes involved in apical junctions and innate immune responses to *Sp*, particularly type I interferon responses. Together, our results demonstrated that CSE-induced mitochondrial dysfunction may contribute to impaired innate immune responses to *Sp*.

## 1. Introduction

Cigarette smoking is largely associated with development and progression of airway complications, inducing airway epithelial damage, mucus hypersecretion, and airway inflammation [1]. The airway epithelium forms the first line of defense against noxious particles/gases and inhaled pathogens by means of physical and immunochemical barriers [1]. Airway epithelial cells (AECs) actively function to detoxify and remove these stressors by mucociliary function as well as production of host defense proteins [2]. Such active cells are highly dependent on mitochondria as the central regulators of cellular energy [3]. Mitochondrial function not only regulates cellular bioenergetic processes, but may also affect epithelial integrity and innate immune responses during inflammatory conditions [3]. AEC dysfunction has been observed in response to cigarette smoke exposure as evidenced by mucociliary dysfunction, defective remodeling, diminished cell–cell contacts, and impaired mitochondrial function [1,3]. As such, a damaged epithelium with abnormal mitochondrial function contributes to the development and progression of airway diseases [3].

Dysfunctional mitochondria in the lungs and airways of smokers may exhibit altered morphology and abnormal quality control processes such as defects in mitochondrial biogenesis and changes in fission and fusion events as well as mitochondrial-specific autophagy or mitophagy [3]. Similar changes have been observed in AEC in response to cigarette smoke (CS) resulting in the accumulation of damaged mitochondria within the AECs and subsequent cell death [4,5]. These mitochondrial quality control processes are important for cellular homeostasis, as they are essential for regulation of the normal turnover of mitochondria upon stress and damage [6]. Collectively, these findings imply that impaired mitochondrial quality control processes may contribute to the pathogenesis of AEC dysfunction in airway diseases.

Microbial composition of the respiratory tract plays a key role in mucosal immune responses to pathogens [7]. Many factors can affect the composition and distribution of the respiratory microbiota of which CS was shown to increase dissemination of *Streptococcus pneumoniae* (*Sp*) to the lower airways [8]. Ultimately, such imbalance of the airway microbiota further enhances airway inflammations. Moreover, several respiratory bacterial and viral pathogens have been reported to alter mitochondrial function in lung epithelial cells [9,10]. However, the impact of *Sp* infection on mitochondrial function in AECs is incompletely understood. Moreover, while CS exposure was shown to induce mitochondrial dysfunction in AECs [11], the effects of CS on mitochondrial function in AECs upon a bacterial infection with *Sp* is unclear. To extend existing knowledge, in this study we investigated mitochondrial function in AECs by measuring mitochondrial oxidative stress, mitochondrial membrane potential, and mitochondrial quality control processes in an in vitro model of exposure to CSE and *Sp*. Furthermore, we examined potential mechanistic links between CS-induced mitochondrial dysfunction and altered innate immune response to *Sp* infection in AECs by looking into pathways that directly or indirectly link the innate immune response to the pathogen with mitochondrial function. 

## 2. Materials and Methods

### 2.1. Cell Culture

A 16HBE-14o- SV-40 immortalized bronchial epithelial cell line was kindly donated by Professor Dieter Gruenert, University of California. The cells were cultivated in T75 flask (Greiner Bio-One GmbH, Frickenhausen, Germany) in minimum essential medium with earle’s salt (Gibco, Life Technologies, Paisley, UK) supplemented with 10% fetal bovine serum (PAN-Biotech GmbH, Aidenbach, Germany), L-Glutamine 2 mM (Invitrogen, Thermo Fisher Scientific, Waltham, MA, USA) and 1% penicillin/streptomycin (Invitrogen, Thermo Fisher Scientific, Waltham, MA, USA) and kept in a humidified incubator at 37 °C with 7.5% CO_2_. Upon 90% confluency, the cells were detached and harvested with Trypsin/EDTA 0.025% (Invitrogen, Thermo Fisher Scientific, Waltham, MA, USA). 

### 2.2. Cigarette Smoke Extract (CSE) Preparation

For preparation of aqueous CSE, 3R4F research cigarettes (University of Kentucky, Lexington, KY, USA) were purchased and used throughout the study. Filterless cigarettes were attached to 1 mL pipette tips and inserted to a 100 mL gas washing bottle (Borosilicate glass, VWR international GmbH, Darmstadt, Germany). Two cigarettes were bubbled through the medium using a peristaltic pump (Longer pump, BT100-2J, Longer Precision Pump Co., Ltd., Hebei, China) at flow rate 180 mL/min (each cigarette combusted in around 5 min), which subsequently was considered as 100% concentration and further diluted with culture medium upon the experiments. The prepared CSE was filtered through a 0.22 µM filter (Merck KGaA, Darmstadt, Germany) to remove particles and pathogens. The aqueous CSE was prepared freshly upon each experiment respectively and used not more than 30 min after preparation.

### 2.3. Streptococcus Pneumoniae Culture

*Sp* clinical isolate 19F was plated on Columbia blood agar (BD Biosciences, San Jose, CA, USA) and kept at 37 °C with 5% CO_2_ overnight. The single colonies were removed by a sterile cotton swab and transferred to autoclaved pre-warmed Todd Hewitt broth medium (Sigma-Aldrich GmbH, Taufkirchen, Germany) supplemented with Bacto Yeast extract (BD Biosciences, San Jose, CA, USA). The initial optical density (OD) of the inoculated broth was measured with a spectrophotometer (GeneQuant, Amersham Biosciences, Freiburg, Germany) and set at ~0.09. The bacteria were grown until mid-log phase point equivalent to an OD_600_ of 0.2–0.3 in the generated growth curve (Figure S1). The multiplicity of infection (MOI) was adjusted based on the repetitive measurement of colony formation unit (CFU) per ml of mid-log phase *Sp* stock. Before each experiment, the bacterial stock was prepared separately, by washing the bacterial stock three times in cold 1X PBS (Gibco, Life Technologies, Paisley, UK) and centrifuging at 14,000× *g* at 4 °C. The bacterial pellets were then resuspended in 1 mL antibiotic-free MEM. 

### 2.4. Experimental Design

16HBE-14o^−^ cells were stimulated with different concentrations of CSE for 3 h, 14 h, and 24 h. Furthermore, the cells were incubated with CSE for 24 h followed by *Sp* infection with MOI 10 and 20 to mimic in vitro COPD exacerbations. For mitochondrial-targeted therapies, the cells were pre-exposed to CSE for 24 h and subsequently incubated with MitoTEMPO (Sigma-Aldrich GmbH, Taufkirchen, Germany) 50 µM or MOTS-c (Eurogentec GmbH, Köln, Germany) 25 µM for 4 h.

### 2.5. MTT Assay

3-(4,5-Dimethyl-2-thiazolyl)-2,5-diphenyl-2*H*-tetrazoliumbromids (MTT) (Sigma-Aldrich GmbH, Taufkirchen, Germany) assay was performed to determine the optimal concentration of CSE which exerts maximal effects on mitochondrial activity in 16HBE cells. MTT was diluted in 1X PBS and filter-sterilized using 0.22 µm filter strip. 16HBE cells were seeded into a transparent 96-well at 10^4^ density and placed into a humidified with 7.5% CO_2_ incubator until forming a confluent monolayer. Subsequently, the cells were stimulated with different concentrations of CSE (5–20%) for 24 h. After removing the medium, diluted MTT with final concentrations of 0.5 mg/mL was added to the cells in a phenol-red-free minimum essential medium (MEM). Following 4 h incubation in the incubator in dark, the insoluble formazan produced by viable cells turned to a soluble solution using cell-grade dimethyl sulfoxide (DMSO) (Carl Roth, Karlsruhe, Germany). The absorbance was read in each well at 560 nm wavelength using a microplate reader (BioTek HT, BioTek Instrument GmbH, Bad Friedrichshall, Germany).

### 2.6. RealTime-Glo MT Assay

RealTime-Glo MT assay (Promega GmbH, Walldorf, Germany) was performed to examine the impact of CSE on the proliferation of metabolically active cells. The 16HBE cells were first seeded in different densities (5 × 10^3^, 10^4^ and 2 × 10^4^ cells/well) in the 96-well white-walled and transparent bottom plate (Greiner Bio-One GmbH, Frickenhausen, Germany) to achieve an optimal cell density in which the signal remains linear throughout the experiment. Next, the cells were seeded at 10^4^ and incubated with 2X RealTime-Glo reagent. The luminescence at assay start (0 h) was then measured with a microplate reader (BioTek HT, BioTek Instrument GmbH, Bad Friedrichshall, Germany). In the next rounds, following incubating of the cells with the reagent the cells were stimulated with medium control or different concentrations of CSE (5–20%) and incubated in at 37 °C with 7.5% CO_2_. The luminescence was measured at 1 h, 3 h, 7 h, and 19 h post-treatment in the plate. The luminescent signal was plotted versus cell number using a linear curve fit in GraphPad prism version 9.3.1.

### 2.7. Intracellular Reactive Oxygen Species (ROS) Levels

Intracellular oxidative stress was evaluated based on detection of fluorescent dye 2′,7′-dichlorodihydrofluorescein diacetate (DCFDA) (DCFDA cellular ROS detection kit, Abcam, Cambridge, UK) using a microplate reader (BioTek HT, BioTek Instrument GmbH, Bad Friedrichshall, Germany). The 16HBE cells were seeded at 10^4^ cells/well density into a 96-well black-walled plate with transparent bottom (Greiner Bio-One GmbH, Frickenhausen, Germany) and incubated for 3 days at the humidified incubator with 7.5% CO_2_. Next, the cells were washed once with diluted assay buffer and stained with 25 µM of DCFDA diluted in an assay buffer for 45 min. The cells were then stimulated with different concentrations of CSE (5–20%), medium control or a ROS-positive control compound tBHP, diluted in the complete serum-free medium. The fluorescent signal was initially measured at 0 h time before stimulating the cells by exciting at 488 nm and emitting at 528 nm (FITC filter set) and a PMT gain set at 45. Afterwards, the plate kept in the incubator and read at 1 h, 3 h, 6 h, 19 h, 21 h, 23 h, 25 h, and 28 h post treatment. The fluorescent signal was calculated by subtracting the unstained background and normalizing the signals from each condition to 0 h. 

### 2.8. FACS Staining for MitoSOX

The mitochondrial ROS levels were measured by MitoSOX^TM^ Red fluorescent-based labelling of the superoxide (Thermo Fisher Scientific, Waltham, MA, USA) generated in the mitochondria of 16HBE cells and, subsequently, by detection of signal with flow cytometry. The cells were seeded into 6-well plates (Corning, Saint Louis, MO, USA) at 3 × 10^5^ cells/well and incubated with medium control or different concentrations of CSE (5–20%) and placed in the humidified incubator (7.5% CO_2_) until 90% confluency. The antibiotic-supplemented medium was replaced with antibiotic-free medium 24 h before the experiment to rule out the impact of antibiotic on pneumococci growth. Next, the stimulated cells were harvested by trypsin/EDTA and washed once with 1X PBS. The cells were then stained with 5 µM of diluted MitoSOX dye (5 mM stock in DMSO) in a serum- and phenol red-free medium for 15 min. Subsequently, the cells were washed three times with 1X PBS and stained with a live/dead fluorescent dye (efluor 780, Thermo Fisher Scientific, Waltham, MA, USA) to gate out dead cells. The fluorescent signal was detected with the 8-colors FACS Canto II device (BD Biosciences, San Jose, CA, USA). The MitoSOX-positive cell population and the shift in fluorescent signal was analyzed using FlowJo software version 10.3. The mean fluorescent intensity of MitoSOX positive cells was plotted for each condition using GraphPad prism version 9.3.1.

### 2.9. Mitochondrial Membrane Potential Analysis

JC-1 dye (MitoProbe JC-1 Assay kit, Thermo Fisher Scientific, Waltham, MA, USA), which shows potential-dependent localization into the mitochondrial membrane, was used to assess the impacts of CSE and *Sp* on the mitochondrial membrane potential. 16HBE cells were plated at 10^4^ cell/well in a black-wall 96-well plate (Greiner Bio-One GmbH, Frickenhausen). After 3 days, the medium was removed, and the cells were incubated in 2 µM JC-1 dye diluted in serum-free medium for 1 h. After measuring the signal from J-aggregates (ref) and monomer (green) at 0 h, the cells were stimulated with medium control or CSE (5–20%), different MOI of *Sp* (MOI 10 and 20) and carbonyl cyanide-p-trifluoromethoxyphenylhydrazone (FCCP) 50 µM (Sigma-Aldrich GmbH, Taufkirchen, Germany) as mitochondrial complex V uncoupler. The fluorescent signal was measured at 1 h, 3 h, 6 h, 19 h, 21 h, 23 h, 25 h, and 27 h post-treatment by exciting the cells at 488 nm and emitting at 529 nm and 590 nm for monomer and J-aggregates, respectively, using the microplate reader (BioTek HT, BioTek Instrument GmbH, Bad Friedrichshall, Germany). The ratio of J-aggregates to green monomer, which is used to calculate the mitochondrial membrane potential, was measured. After subtraction of the background, the ratio of red/green fluorescent signal at each time point was normalized to 0 h and plotted versus time.

### 2.10. Immunoblotting

16HBE cells were seeded into 10 cm culture dishes (Corning, Saint Louis, MO, USA) and placed in the humidified incubator until reaching 90% confluency. Based on the experimental design, the cells were either stimulated with medium control and CSE for 3 h, 14 h, and 24 h or stimulated with CSE 28 h, *Sp* MOI 10, and MOI 20 for 4 h, CSE 24 h followed by *Sp* MOI 10 and 20 for 4 h as well as CSE 24 h followed by 4 h medium, and FCCP 50 µM for 4 h. The cells were lysed with a 1X-chilled RIPA buffer (RIPA buffer 10×, Cell Signaling Technology, Leiden, Netherlands) containing the EDTA-free protease inhibitor cocktail (cOmplete ^TM^ Ultra Mini tablet, EDTA-free, Roche, Penzberg, Germany) and after centrifugation for 10 min at 14,000× *g*, the supernatants were collected and kept at –80 °C until further use.

Mitochondrial fractions were isolated from the cells using a commercial kit (Mitochondrial Fractionation kit, Active Motif, Carlsbad, CA, USA) by gradient centrifugation. Accordingly, 2 × 10^6^ 16HBE cells were seeded into the culture dishes and placed in the humidified incubator until reaching 90% confluency. For each condition, cells from three cell culture dishes were used by pulling cell pellets together to harvest 3 × 10^7^ cells. Next, the cells were washed with cold 1X PBS and collected by gently scratching the culture surface in chilled 1X PBS using cell scraper (Greiner Bio-One GmbH, Frickenhausen). Following cell lysis with a pestle homogenizer (Carl Roth, Karlsruhe, Germany), debris and intact cells were separated by centrifugation at 800 g. Next, the lysates were centrifuged at 10,000 g to pellet the mitochondria. The isolated mitochondria were lysed in the mitochondrial buffer including a protease inhibitor and DTT (Mitochondrial Fractionation kit Active Motif, Carlsbad, CA, USA) and were then aliquoted and transferred to –80 °C.

The immunoblotting was performed to evaluate the expression of selected mitochondrial targets in both total cell lysates and the cytosolic or mitochondrial fractions. The protein concentrations in each cell lysate were measured using a BCA kit following the manufacturer’s instruction (Thermo Fisher Scientific, Waltham, MA, USA). After calculating and normalizing the protein concentration, the lysates were diluted in a 1× sample buffer (1% β-Mercaptoethanol in 4X Laemmli buffer, BioRad, Feldkirchen, Germany) and boiled at 95 °C for 5 min and transferred to –80 °C. Immunoblotting was conducted using two different protocols, respectively, *i* and *ii*. The samples (4.25–10 µg) were loaded together with *i* one protein ladder (PageRuler, Thermo Fisher Scientific, Waltham, MA, USA) or *ii* at least two protein ladders (Precision Plus Protein^TM^ All Blue Standards #161-0373, BioRad, Feldkirchen, Germany) into *i* manually casted SDS-PAGE gels or *ii* Criterion XT Precast 4–12% or 12% Bis-Tris gels (BioRad, Feldkirchen, Germany). Subsequently, the proteins were separated by electrophoresis at 100–130 for respectively *i* 2.5 h (Mini-Protean tetracell, BioRad, Feldkirchen, Germany) or *ii* 1 h (BioRad, Feldkirchen, Germany). The proteins on the gels were then transferred to the membrane by electroblotting to *i* the methanol-activated PVDF membrane (0.45 µm; Merck KGaA, Darmstadt, Germany) using a semi-dry transfer system (Semi-dry Blotter, Maxi, Carl Roth, Karlsruhe, Germany) or *ii* the nitrocellulose transfer membrane (0.45 µm; BioRad, Feldkirchen, Germany) using a Bio-Rad Criterion Blotter. In case of protocol *ii*, the Nitrocellulose membranes were incubated for 5 min with 0.2% (*w*/*v*) Ponceau S in 1% (*v*/*v*) acetic acid (Sigma-Aldrich GmbH, Taufkirchen, Germany) followed by MilliQ wash and imaging, conducted to quantify the total protein content for normalization, using the Amersham^TM^ Imager 600 (GE Healthcare, Solingen, Germany). Subsequently, the membranes were washed, and non-specific binding sites were blocked by 1 h incubation in milk in a Tween20 Tris-buffered saline (TBST) buffer *i* 5% skimmed milk (Carl Roth, Karlsruhe, Germany) in TBST (Tris-base 200 mM, NaCl 1.4 M, 0.1% *v/v* Tween20) or *ii* 3% non-fat dry milk (Campina Melk Unie, Eindhoven, The Netherlands) in TBST (Tris-base 20 mM, NaCl 137 mM, 0.1% (*v*/*v*) Tween20). Next, the membranes were washed and incubated in the primary target-specific antibodies (Table S1) diluted in 3% Bovine Serum Albumin (BSA fraction V, GE Healthcare, Solingen, Germany) or non-fat dry milk in TBST at 4 °C overnight. Following a washing step, the membranes were incubated in horseradish peroxidase-conjugated secondary antibodies (Table S1) diluted in either 3% BSA, 3% or 5% milk (Campina or Carl Roth) in TBST for 1 h at room temperature. The membranes were finally washed with TBST before imaging. The membranes were incubated for 1–3 min with *i* the chemiluminescent substrate Pierce^TM^ ECL plus (Thermo Fisher Scientific, Waltham, MA, USA) and visualized using a CCD imager (Intas ChemoCam, INTAS Science Imaging, Göttingen, Germany) or *ii* a 0.25× Supersignal West FEMTO or 0.5× Supersignal West PICO chemiluminescent substrate (Thermo Fisher Scientific, Waltham, MA, USA) and visualized using the Amersham™ Imager 600 (GE Healthcare, Solingen, Germany).

The abundance of the target proteins was quantified and normalized using *i* ImageJ software and normalized to loading control proteins GAPDH or β-actin or *ii* Image Quant software (GE Healthcare, Solingen, Germany) and the total protein loading content was assessed by Ponceau S staining over the entire size range of proteins (10 kDa–250 kDa). Selected Western blot images of one sample/treatment group are reflecting changes for the replicates/experiment. Representative Western blot images shown in the figures of this manuscript have been equally adjusted for brightness and contrast throughout the picture. 

### 2.11. Immunofluorescence Staining

The cells were seeded into the FluroDish (WPI, Saratosa, FL, USA) at 15 × 10^4^ cells/dish and stimulated with either CSE for 28 h or with CSE for 24 h followed by MitoTEMPO 50 µM for 4 h (Sigma-Aldrich GmbH, Taufkirchen, Germany) or MOTS-c 25 µM (Eurogentec GmbH, Köln, Germany). The cells were then fixed with pure chilled methanol. After washing, the cells were permeabilized with 5% goat serum (Cell Signaling Technology, Leiden, Netherlands) and 0.01% TritonX-100 (Carl Roth, Karlsruhe, Germany) in 1X PBS for 1 h. Subsequently, the cells were incubated in primary antibodies DRP1, p-DRP1, TOMM20, ERRα, ZO-1, and E-cadherin (Table S2), diluted in 1% BSA with 0.01% TritonX-100 in 1X PBS, and kept at 4 °C overnight. Following washing with 5% goat serum (Cell Signaling Technology, Leiden, The Netherlands) in 1X PBS, the cells were incubated in the secondary antibodies (goat anti-rabbit Alexa fluor 647, goat anti- mouse Alexa fluor 488, Cell Signaling Technology, Leiden, Netherlands) for 1 h. After the final washing step, the cells were stained with 4′,6-diamidino-2-phenylindole (DAPI) (Sigma-Aldrich GmbH, Taufkirchen, Germany) to locate the nucleus. The cells were then mounted with a mounting medium (ProTaqs^®^ MountFlour, quartet, Berlin, Germany), covered with coverslips and placed at 4 °C overnight. Microscopy was performed under a fluorescent microscope Zeiss Axiovert 200 m (Carl Zeiss MicroImaging GmbH, Oberkochen, Germany) with the appropriate filter sets. The images were analyzed using the latest version of ImageJ software. 

### 2.12. Mitochondrial Morphology (TEM Analysis)

The cells were prepared as mentioned above and fixed in six well plates by adding the fixative solution, a 0.1 M EM-HEPES buffer (HEPES 0.1 M, 0.09 M sucrose, 10 mM CaCl_2_, 10 mM MgCl_2_, pH 6.9) with 5% paraformaldehyde and 2% glutaraldehyde. The fixed samples were washed twice with a 0.1 M EM-HEPES buffer and treated with osmium tetroxide (1% in HEPES buffer) for 1 h at room temperature. After additional washing steps with the HEPES buffer, the cells were mechanically detached from the surface and centrifuged in a swingout rotor for 4 min at 5000 RPM. The pellet was stabilized in 2% noble agar before dehydration in a graded series of ethanol (10%, 30%, 50%, 70%, and 90%) on ice, and two steps in 100% ethanol at room temperature, each step for 30 min. Samples were subsequently infiltrated with the LR White (LRW) resin (LRW: EtOH: 1:1, 2:1, 2 × 100%), each incubating step for approx. 8 h respectively overnight followed by a polymerization step in small gelatin capsules at 50 °C for 48 h. Ultrathin sections of approx. 50–70 nm thickness were prepared with an Ultramicrotome Ultracut UC7 (Leica, Wetzlar, Germany) and counterstained with 4% aqueous uranyl acetate for 3 min and both with and without lead citrate for 15 s. The TEM image acquisition at calibrated magnifications was performed with a Libra 120 Plus (Zeiss, Oberkochen, Germany) using an acceleration voltage of 120 kV. For image analysis, the software ITEM (Olympus) was used.

### 2.13. Gene Expression Microarray

RNA from 16HBE cells was isolated and purified using the RNeasy Mini plus and RNase-free DNase kit (both Qiagen, Hilden, Germany). For each stated condition, two independent biological replicates were analyzed. Samples were amplified, labelled, fragmented, and hybridized to human ClariomTM S microarray (Thermo Fisher Scientific, Waltham, MA, USA) according to manufacturer’s instructions. Microarray scanning was performed using a GeneChipTM 3000 scanner and GCOSv1.1 software. Microarray analysis was performed at the Genome Analytics Group at the Helmholtz Centre for Infection Research, Braunschweig (Germany). Data analyses were conducted using Transcriptome Analysis Console 4.0 (Thermo Fisher Scientific, Waltham, MA, USA). Data were normalized with the Signal Space Transformation Robust Multi-Array Analysis (SST-RMA) algorithm with quantile normalization, log2-transformation of signal intensities (SI). Only transcripts with SI-values above the 20th percentile (4.5) of the total normalized SI-distribution in all microarrays from all conditions were retained. Fold changes of transcripts were calculated from mean SI of replicate microarrays versus the untreated medium control. Only transcripts with an absolute fold change > 3-fold in at least one condition were retained. Significance of differential expression was calculated by ANOVA. Only transcripts with an ANOVA *p*-value < 0.05 were retained. Normalized log2 SI data of regulated transcripts were z-score-transformed, k-means-clustered, color-coded, and visualized using Genesis Software (Version 1.8.1) [12]. 

Gene Set Enrichment Analysis (GSEA) of each condition in reference to the unstimulated medium control with a fold-change-based gene ranking was performed using the GSEA desktop application (Version 4.1.0) that is available online [13,14]. Gene sets from GSEA-Hallmark, Reactome, and Gene Ontology database were used. Only gene sets with FDR < 0.1 were considered. 

### 2.14. Statistical Analysis

Statistical analysis and graphical representation of the data were done using GraphPad prism version 9.3.1. All experiments were repeated at least twice with different passages of the cells, and the calculations were performed based on these repeated measurements and plotted as mean ± SEM. Dependent on the experimental condition, the significant difference between the conditions were determined using one-way and two-way ANOVA with Dunnett’s multiple comparison test or paired a two-tailed *t*-test. Statistical significance was considered if *p*-values were less than 0.05 (* *p* < 0.05), 0.001 (** *p* < 0.001), and 0.0001 (*** *p* < 0.0001). 

## 3. Results

### 3.1. Optimization of the CSE-Induced Mitochondrial Dysfunction Model

In order to establish our in vitro model of CSE-induced mitochondrial dysfunction and to determine the optimal CSE concentration to be used in our study, 16HBE cells were cultured for 24 h in the presence of graded concentrations of CSE (5–20%) and the levels of cell proliferation, mitochondrial reactive oxygen species (mtROS) and mitochondrial membrane potential (MMP) were measured (Figure S2).The results show that the effects of CSE on mitochondrial function is concentration- and time-dependent. Furthermore, the highest analyzed CSE concentration (20%) not only impairs mitochondrial function, but also has a negative effect on cell proliferation and viability. As such, 15% of CSE was selected for subsequent experiments and for the sake of simplicity, we will use the abbreviation CSE without further stating the percentage of 15% in the following paragraphs.

### 3.2. CSE Reduces Protein Expression of Key Regulators Involved in Mitochondrial Function in 16HBE Cells

Immunoblotting was performed to check the impact of CSE not only on the expression of different mitochondrial complex proteins, but also to investigate the expression of mitochondrial outer membrane proteins and key regulators of mitochondrial biogenesis. 

**Figure 1:**
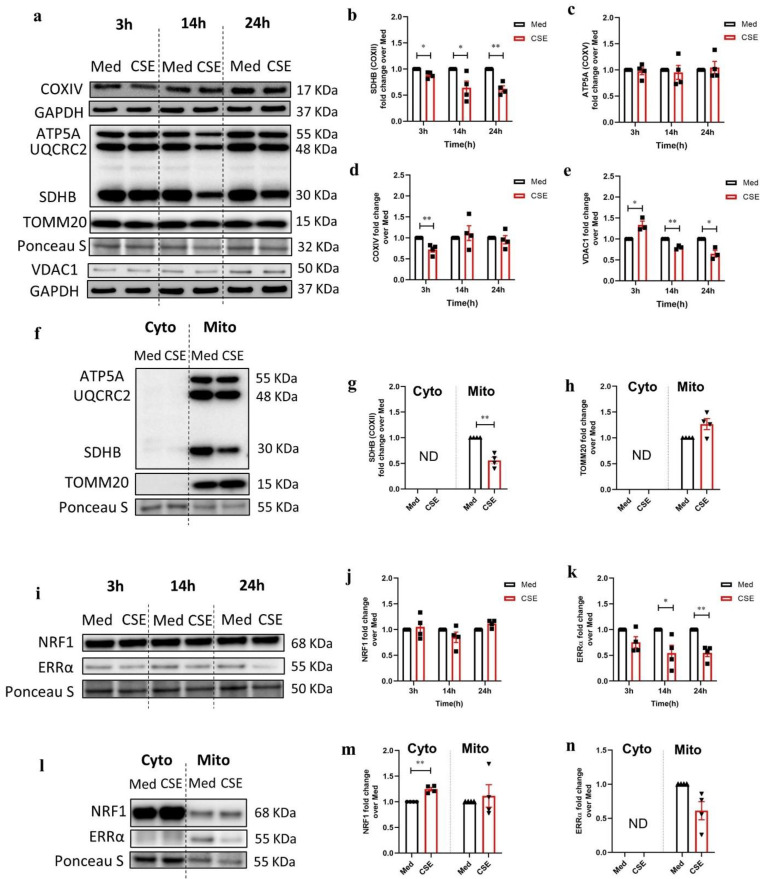
Abundance of mitochondrial complex (OXPHOS) components and constituents controlling mitochondrial biogenesis upon CSE stimulation in 16HBE cells. (**a**) Immunoblot representing protein expression of subunits of mitochondrial oxidative phosphorylation (OXPHOS) complexes: complex II (succinate dehydrogenase B-SDHB), complex III (cytochrome b-c1 complex subunit 2-UQCRC2), complex IV (cytochrome c oxidase subunit IV, COXIV), and complex V (ATP synthase subunit alpha-ATP5A); and translocase of the outer mitochondrial membrane complex subunit 20 (TOMM20) and voltage-dependent anion channel (VDAC1) in the total cell lysates of 16HBE cells incubated with medium (Med) as control or cigarette smoke extract (CSE) for 3 h, 14 h, and 24 h. Fold change of protein levels of (**b**) SDHB, (**c**) ATP5A, (**d**) COXIV, and (**e**) VDAC1 in the total cell lysates. The data are represented as mean ± SEM of four independent experiments (N = 4). (**f**) Immunoblots representing subunits of mitochondrial OXPHOS complex proteins in the cytoplasmic (Cyto) and mitochondrial fractions (Mito) of 16HBE cells incubated with Med or CSE for 24 h. Fold change of SDHB protein expression of (**g**) SDHB and (**h**) TOMM20 compared to the medium control (N = 4). (**i**) Immunoblots representing the protein expression of constituents involved in mitochondrial biogenesis including nuclear respiratory factor 1 (NRF1) and estrogen- related receptor α (ERRα) in the total cell lysates of 16HBE cells upon incubation with medium as a control or CSE for 3 h, 14 h, and 24 h. Fold change of protein levels of (**j**) NRF1 and (**k**) ERRα compared to the medium control (N = 4). (**l**) Immunoblots representing NRF1 and ERRα in the Cyto and Mito fractions upon incubation of 16HBE cells with either medium or CSE for 24 h. Fold change of protein levels of (**m**) NRF1 protein expression and (**n**) ERRα compared to the medium control (N = 4). The data are represented as mean ± SEM of four independent experiments (N = 4). Representative Western blot images are selected and depicted of one replicate as quantified in the corresponding graph. Normalization of protein expression was performed by GAPDH or Ponceau S (Pon S) staining. Statistically significant differences were calculated with multiple *t*-test and with Holm–Sidak post hoc correction test (* *p* < 0.05, ** *p* < 0.001). ND: not detectable.

Protein levels of several subunits of oxidative phosphorylation (OXPHOS) complexes were initially probed in total cell lysates of 16HBE cells stimulated with CSE for 3 h, 14 h, and 24 h (Figure 1a). Immunoblotting revealed a significant reduction in the protein expression of a subunit of complex II protein (succinate dehydrogenase B-SDHB) after 3 h, 14 h, and 24 h stimulation with CSE (Figure 1b), while the expression of subunits of complex III (cytochrome b-c1 complex subunit 2-UQCRC2) and complex V (ATP synthase subunit alpha-ATP5A) proteins as well as a mitochondrial import protein, translocase of the outer mitochondrial membrane complex subunit 20 (TOMM20) remained unchanged (Figure 1a,c; quantification not shown for UQCRC2 and TOMM20) at the same time point. Furthermore, CSE exposure for 3 h resulted in significantly decreased complex IV cytochrome c oxidase (subunit of COXIV) protein levels; however, it returned to basal levels already after 14 h and remained unaltered after 24 h (Figure 1d). Although 3 h stimulation with CSE increased the protein levels of mitochondrial outer membrane protein voltage-dependent anion channel 1 (VDAC1), 14 h and 24 h of treatment with CSE significantly decreased the protein expression of VDAC1 compared to the medium (Figure 1a,e). 

In addition, the mitochondrial proteins were separately probed in the cytoplasmic and mitochondrial fractions upon stimulation of 16HBE cells with CSE for 24 h (Figure 1f). The abundance of SDHB (complex II) significantly decreased upon stimulation with CSE for 24 h in the mitochondrial fractions (Figure 1g). Moreover, the abundance of TOMM20 slightly increased but failed to reach statistically significant difference compared to the medium (*p* = 0.08) (Figure 1h).

Mitochondrial biogenesis was probed by quantifying the expression of proteins essentially involved in the molecular control of this process, including nuclear respiratory factor 1 (NRF1) and estrogen-related receptor α (ERRα) in the total 16HBE cell lysates (Figure 1i). The abundance of the NRF1 protein was unaltered post-CSE exposure compared to the medium control (Figure 1j). ERRα protein levels were reduced both 14 h and 24 h post-CSE (Figure 1k). Moreover, NRF1 and ERRα were analyzed in the cytoplasmic and mitochondrial fractions of 16HBE cells exposed to CSE for 24 h (Figure 1l). The levels of NRF1 significantly increased in the cytoplasmic fraction upon CSE for 24 h (Figure 1m), while ERRα was only detected in the mitochondrial fraction and decreased but not significantly (Figure 1n). Furthermore, immunostaining for ERRα revealed an increase in peri-nuclear accumulation of the ERRα protein when the cells were stimulated with CSE for 28 h (Figure S3). The peri-nuclear accumulation of ERRα, induced by CSE, was not altered by an additional 4 h post-treatment with 25 µM MOTS-c, a mitochondrial-derived peptide regulating OXPHOS. Stimulation with 50 µM FCCP increased the spread of the fragmented ERRα signal in the cytoplasm. Thus, ERRα peri-nuclear localization in 16HBE cells seems specific for CSE treatment.

Together, these data show that CSE significantly reduces the abundance of subunits of OXPHOS complexes in 16HBE cells. Furthermore, while CSE reduced the membrane potential-dependent protein VDAC1, CSE increased the membrane import protein TOMM20. The ERRα protein, which is essential for mitochondrial biogenesis, was reduced in 16HBE cells upon CSE stimulation and showed a distinct peri-nuclear localization. 

### 3.3. CSE Affects the Abundance of Regulators Associated with Mitochondrial Quality Control Processes

**Figure 2:**
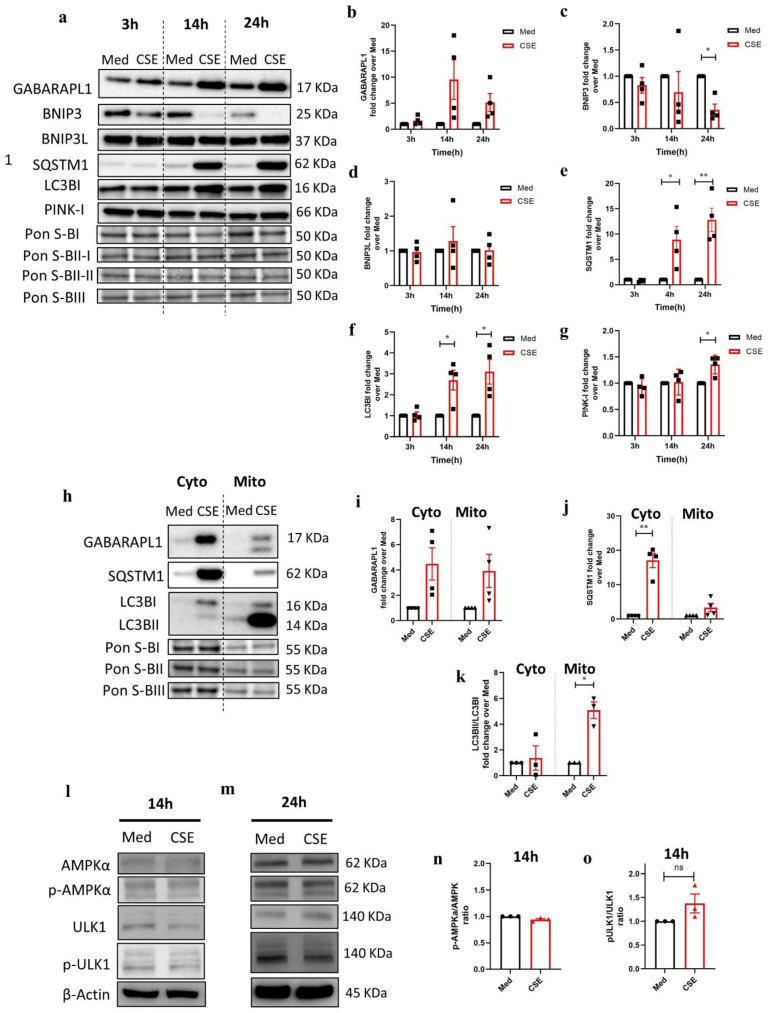
Abundance of key regulators involved in mitochondrial-specific autophagy (mitophagy) in 16HBE cells upon stimulation with cigarette smoke extract. (**a**) Immunoblots representing protein expression of receptor-mediated constituents gamma-aminobutyric acid A receptor-associated protein-like 1 (GABARAPL1), BCL2-interacting protein 3 (BNIP3), BCL2-interacting protein 3-like (BNIP3L), and ubiquitin-mediated mitophagy factor phosphatase and tensin homolog (PTEN)-induced kinase I (PINK-I) mitophagy factors as well as mitophagy adaptor proteins sequestosome-1 (SQSTM1) and (ratio) microtubule-associated protein 1 light chain 3 beta (LC3B)I/II in 16HBE cells incubated with cigarette smoke extract (CSE) or medium (Med) for 3 h, 14 h, and 24 h in total cell lysates. Fold change of protein levels of (**b**) GABARAPL1, (**c**) BNIP3, (**d**) BNIP3L, (**e**) SQSTM1, (**f**) LC3BI, and (**g**) PINK-I over the medium control. The data are represented as mean ± SEM of four independent experiments (N = 4). (**h**) Immunoblots representing the selected mitophagy factors in the cytosolic (Cyto) and mitochondrial (Mito) fraction of the cells stimulated with either Med or CSE for 24 h. Fold change of protein levels of (**i**) GABARAPL1, (**j**) SQSTM1, and (**k**) LC3BII/LC3BI ratio in CSE-stimulated cells over the Med control. The data are represented as mean ± SEM of four independent experiments with triplicate samples (N = 4). Immunoblotting of adenosine monophosphate (AMP)-activated protein kinase (AMPK)α, phospho-AMPKα (Thr172), unc-51-like autophagy-activating kinase 1 (ULK1), phospho-ULK1 (Ser555) upon incubation with either Med or CSE for (**l**) 14 h and (**m**) 24 h. Fold change of protein levels of (**n**) p-AMPKα/AMPKα ratio and (**o**) p-ULK-1/ULK1 ratio upon incubation with medium or CSE for 14 h. The data are represented as mean ± SEM of three independent experiments with triplicate samples (N = 3). Normalization of protein expression was performed using Ponceau S (Pon S) staining (GABARAPL1 to Pon S in blot I, BNIP3 and LC3B to Pon S blot II-I, BNIP3L and PINK-I to Pon S blot II-II, SQSTM1 normalized to Pon S in blot III) or β-actin. Representative Western blot images are selected and depicted of one replicate representing the changes of all replicates as quantified in the corresponding graph. Significant differences were analyzed by a paired two-tailed *t*-test (* *p* < 0.05, ** *p* < 0.001).

**Figure 3:**
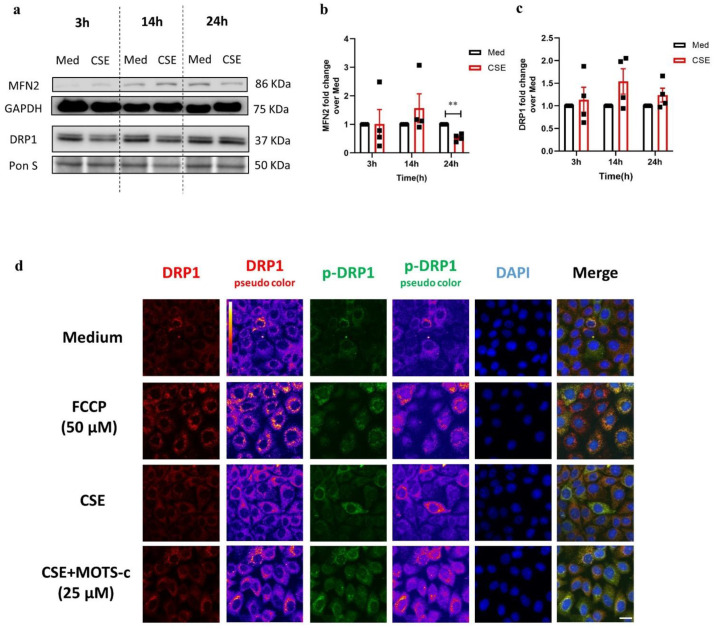
Stimulation with cigarette smoke affects expression of mitochondrial fission/fusion-associated proteins. (**a**) Immunoblots representing protein expression of fusion-marker mitofusion 2 (MFN2) and fission-marker dynamin-related protein 1 (DRP1) in 16HBE cells incubated with medium (Med) or cigarette smoke extract (CSE) for 3 h, 14 h, and 24 h. Fold change of protein levels of (**b**) MFN2 and (**c**) DRP1 in CSE-stimulated conditions over the medium control. The data are represented as mean ± SEM of four independent experiments with triplicate samples (N = 4). Normalization of protein expression was performed using GAPDH or Ponceau S (Pon S) staining. Representative Western blot images are selected and depicted of one replicate representing the changes of all replicates as quantified in the corresponding graph. Significant differences were calculated by multiple *t*-test and with the Holm–Sidak post hoc correction test (** *p* < 0.001). (**d**) Immunofluorescence imaging of treated cell co-stained with anti-DRP1 (red) and anti-phospho-DRP 1 at serine 616 (green) antibodies in combination with 4′,6-diamidino-2-phenylindole (DAPI) nuclear staining in blue. 16HBE cells were either incubated with for 28 h with medium and CSE or CSE for 24 h followed by 4 h treatment with 25 µM MOTS-c and 24 h medium, with 4 h carbonyl cyanide-p-trifluoromethoxyphenylhydrazone (FCCP). False color-coded image columns are shown here to depict differences in staining intensity. Merged images have been gamma-corrected to visualize weak signals without losing the highlights. The scale bar is equivalent to 20 µm.

Mitochondrial quality control processes are crucial for maintaining mitochondrial homeostasis especially upon damage by, e.g., exogenous stimuli. In order to assess the influence of CSE on these processes, the proteins involved in mitophagy and mitochondrial fission and fusion were probed in total cell lysates by immunoblotting (Figure 2a and Figure 3a). Protein expression of a constituent involved in receptor-mediated mitophagy and autophagophore formation, gamma-aminobutyric acid (GABA) A receptor-associated protein-like 1 (GABARAPL1), increased in the total cell lysates after 14 h and 24 h of stimulation with CSE, but failed to reach a statistically significant difference compared to the medium control (*p* = 0.06 and *p* = 0.05) (Figure 2b). In contrast, the levels of another protein involved in receptor-mediated mitophagy and cell death BCL2-interacting protein 3 (BNIP3) were significantly reduced after 24 h of exposure of the cells to CSE (Figure 2c). Moreover, the abundance of mitophagy adaptor protein sequestosome-1 (SQSTM1) was increased upon 14 h and 24 h stimulation with CSE compared to the matched medium control (Figure 2e). The protein levels of another adaptor of mitophagy microtubule-associate protein 1 light chain 3 beta (LC3B) I were increased as well, both 14 h and 24 h post CSE stimulation (Figure 2f). The protein levels of the ubiquitin-mediated mitophagy factor, phosphatase, and tensin homolog (PTEN)-induced putative kinase I (PINK-I) were significantly increased in the total cell lysates of 16HBE cells stimulated with CSE for 24 h as compared to the medium control (Figure 2g). 

Furthermore, these mitophagy proteins were studied in the cytoplasmic and mitochondrial fractions upon stimulation with medium or CSE for 24 h (Figure 2h). The levels of GABARAPL1 increased in both cytoplasmic and mitochondrial fractions upon CSE 24 h incubation but failed to reach a statistically significant difference compared to the medium (Figure 2i). The abundance of SQSTM1 and the LC3BII/LC3BI ratio were significantly increased in, respectively, the cytosolic and the mitochondrial fraction of CSE-stimulated cells after 24 h compared to the medium control (Figure 2j,k). 

Subsequently, phosphorylation of mitophagy regulators adenosine monophosphate (AMP)-activated protein kinase (AMPK)α at threonine 172 and unc-51-like autophagy activating kinase 1 (ULK1) at serine 555 was assessed using immunoblotting of total cell lysates from 16HBE cells stimulated with CSE for 14 h and 24 h (Figure 2l,m). The ratio of phosphorylated AMPKα to total AMPKα did not alter at 14 h and 24 h post CSE treatment compared to the medium control (Figure 2n, quantification not shown for 24 h). Phosphorylated ULK1 only slightly increased upon 14 h (non-significant) (Figure 2n) and returned to the basal levels 24 h post CSE treatment compared to the medium control (Figure 2o, quantification not shown).

Key constituents of mitochondrial fusion and fission were examined by immunoblotting (Figure 3a). Mitofusion 2 (MFN2), one of the proteins regulating mitochondrial fusion, was significantly reduced in 16HBE cells upon stimulation with CSE for 24 h compared to the medium control (Figure 3b). Moreover, total levels of the fission factor dynamin-related protein 1 (DRP1) protein appeared to be increased after 14 h and 24 h in response to CSE compared to the medium but failed to reach statistical significance (*p* = 0.10 and 0.17, respectively) (Figure 3c). Immunostaining, however, revealed an increase in phosphorylated DRP1 at serine 616 (activated form of the protein) upon CSE as well as in response to 50 µM FCCP (Figure 3d). As depicted in Figure 3d, post-treatment with 25 µM MOTS-c attenuated CSE-induced increase in p-DRP1 and improved DRP1 signals as compared to the CSE-stimulated condition implying that improving mitochondrial OXPHOS may suppress mitochondrial fission. 

Together, these findings indicate that CSE stimulation affects the regulatory pathways controlling mitochondrial function and content including both receptor-mediated and ubiquitin-mediated mitophagy as well as mitochondrial fission and fusion.

### 3.4. CSE-Induced Mitochondrial Dysfunction Affects Epithelial Barrier Integrity

**Figure 4:**
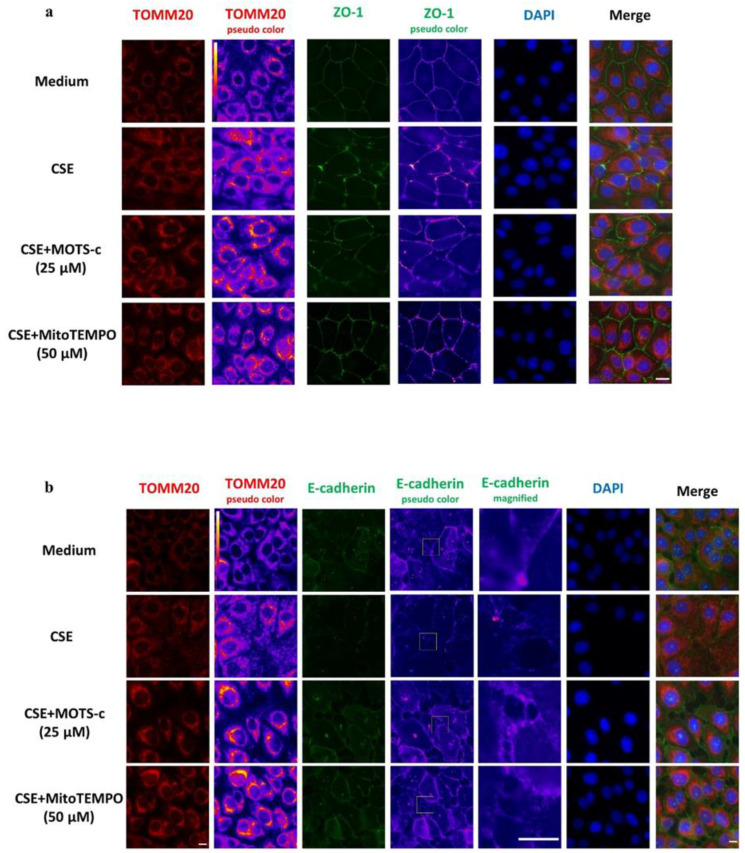
Airway epithelial barrier disruption induced by cigarette smoke extract is reversible with mitochondrial-targeted compounds. 16HBE cells incubated for 28 h with medium (control) or cigarette smoke extract (CSE) for 28 h, or for 24 h with CSE followed by incubation for further 4 h with either mitochondrial-targeted compounds MOTS-c (25 µM) or MitoTEMPO (50 µM). (**a**) Immunofluorescence imaging of treated cells co-stained with antibodies against translocase of the outer mitochondrial membrane complex subunit 20 (TOMM20) in red, the tight junction protein zonula occludens (ZO-1) in green or the nucleus with 4′,6-diamidino-2-phenylindole (DAPI) for nucleus staining (blue). (**b**) Treated cells were (co-)stained with anti-TOMM20-antibody (red) and anti-E-cadherin-antibody (green) or with nuclear staining dye DAPI (blue). False color-coded image columns shown here to depict differences in intensities. To visualize the barrier disruption, magnified regions of interest are indicated in the fourth column and shown in column five. Merged images have been gamma-corrected to visualize weak signals without losing the highlights. The scale bar is equivalent to 20 µm (**a**) and 10 µM (**b**).

In order to unravel the links between CSE-induced mitochondrial dysfunction and epithelial cell junction integrity, mitochondrial-targeted compounds were used to examine whether improving mitochondrial function would affect barrier integrity. To this end, 16HBE cells were stimulated with CSE followed by incubation with the mitochondrial ROS scavenger MitoTEMPO (50 µM) and MOTS-c (25 µM) for 4 h. Methanol-fixed cells were stained for cell junction proteins (ZO-1 and E-cadherin) and the mitochondrial outer membrane protein TOMM20 and subsequently analyzed by immunofluorescence microscopy. CSE stimulation disrupted ZO-1 and E-cadherin, which was partly reversible by MOTS-c treatment (Figure 4a,b). Moreover, incubation with MitoTEMPO for 4 h counteracted the effect of CSE on the barrier function by ameliorating ZO-1 and E-cadherin (Figure 4b).

Together, the findings on physical epithelial barrier function indicate that, while CSE disrupts the cell–cell junctions, post-CSE treatment with mitochondrial-targeted compounds, which improve mitochondrial oxidant status, restores cell junctional defects induced by CSE.

### 3.5. Streptococcus Pneumoniae Infection with Prior CSE Exposure Induces Mitochondrial Dysfunctions and Ultrastructure Damage in 16HBE Cells

In order to assess the impact of (i) *Sp* infection and (ii) *Sp* infection in cells pre-exposed to CSE on mitochondrial read-out parameters, we conducted mtROS and MMP measurements as well as immunoblotting for selected proteins involved in mitochondrial metabolism and mitophagy.

**Figure 5:**
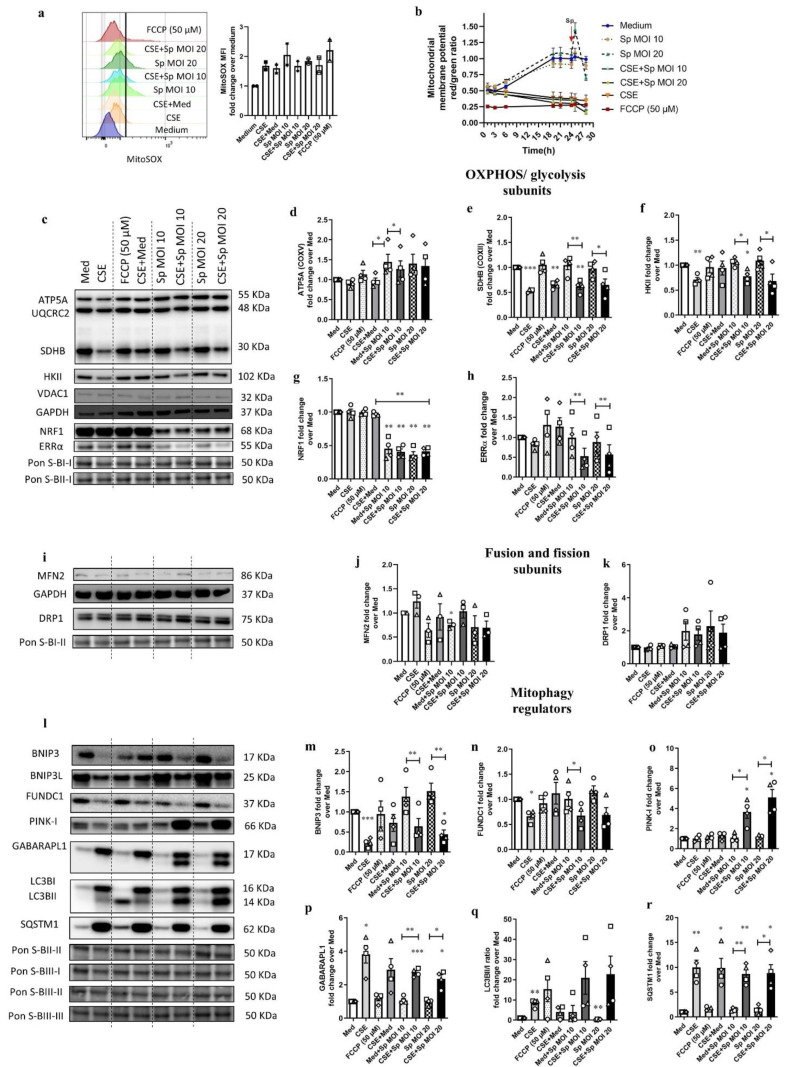
Cigarette smoke extract exposure followed by *Streptococcus pneumoniae* infection induced mitochondrial dysfunction in 16HBE cells. 16HBE cells were incubated with either medium (Med) for 28 h, cigarette smoke extract (CSE) for 28 h, CSE for 24 h followed by medium for 4 h (cessation), medium for 24 h followed by *Streptococcus pneumoniae* (*Sp*) multiplicity of infection (MOI) 10 for 4 h, CSE for 24 h followed by *Sp* MOI 10 for 4 h, medium for 24 h followed by *Sp* MOI 20 for 4 h, CSE 24 h followed by *Sp* MOI 20 for 4 h, and medium for 24 h followed by carbonyl cyanide-p-trifluoromethoxyphenylhydrazone (FCCP) 50 µM for 4 h. (**a**) Half offset histogram showing changes in the curve of MitoSOX fluorescence signal as measured by FACS and analyzed by FlowJo, indicative of changes in mitochondrial ROS levels (**left** panel), and fold change of mean fluorescence intensity (MFI) of MitoSOX-positive cells upon treatments calculated over the medium (**right** panel). The data are represented as mean ± SEM of two independent experiments. (**b**) Mitochondrial membrane potential using JC-1 by dividing J-aggregate (red signal) by monomer (green signal) in the stimulated 16HBE cells normalized to the assay start 0 h. The graph is representative of one out of two independent experiments with similar outcome in triplicates. (**c**) Immunoblots representing protein expression of subunits of oxidative phosphorylation complex, components of the glycolytic pathway, and constituents controlling mitochondrial biogenesis in the total cell lysates upon stimulation with either medium for 28 h, CSE for 28 h or medium for 24 h followed by FCCP 50 µM for 4 h, CSE for 24 h followed by medium for 4 h, *Sp* MOI 10 for 4 h, CSE for 24 h followed by *Sp* MOI 10 for 4 h, medium for 24 h followed by *Sp* MOI 20 for 4 h, and CSE for 24 h followed by *Sp* MOI 20 for 4 h. Densitometrically quantified fold change of protein levels was calculated for (**d**) subunit of complex V (adenosine triphosphate 5A-ATP5A), (**e**) subunit of complex II (succinate dehydrogenase B-SDHB), (**f**) hexokinase II (HKII), (**g**) nuclear respiratory factor 1 (NRF1), as well as (**h**) estrogen related receptor α (ERRα). The data are represented as mean ± SEM from four independent experiments (N = 4). Immunoblotting of total cell lysates from stimulated cells for proteins involved in (**i**) mitochondrial fusion and fission, as well as in (**l**) autophagy and mitophagy. Protein levels of (fold change) (**j**) fusion-associated protein mitofusion 2 (MFN2), (**k**) fission-associated protein dynamin-related protein 1 (DRP1); receptor-mediated mitophagy regulators: (**m**) BCL2-interacting protein 3 (BNIP3), (**n**) Fun14 domain-containing protein 1 (FUNDC1), marker associated with ubiquitin-mediated mitophagy: (**o**) PTEN-induced kinase I (PINK-I); and autophagy-associated constituents: (**p**) gamma-aminobutyric acid (GABA) A receptor-associated protein-like 1 (GABARAPL1), (**q**) ratio of microtubule-associated protein 1 light chain 3 beta (LC3B) II to I (**r**) sequestosome-1 (SQSTM1). The data are represented as mean ± SEM of four independent experiments in triplicates (N = 4). Normalization of protein expression was performed using GAPDH or Ponceau S (Pon S) staining, respectively, for ATP5a, SDHB, and HKII to Pon S blot (B) I-I, DRP1 and SQSTM1 to Pons S BI-II, NRF1 and ERRα to Pon S BII-I, BNIP3 and LC3BI/II to Pon S BII-II, BNIP3L and PINK-I to Pon S BIII-I, FUNDCI to BIII-II and GABARAPL1 to Pons S BIII-I. Representative Western blot images are selected and depicted of one replicate representing the changes of all replicates as quantified in the corresponding graph. The significant differences of treated cells versus medium control were calculated with a paired two-tailed *t*-test (* *p* < 0.05, ** *p* < 0.001, *** *p* < 0.0001).

Firstly, MitoSOX was quantified by FACS to examine the mtROS levels upon *Sp* infection alone (MOI 10 and MOI 20) and *Sp* infection in CSE pre-exposed 16HBE cells. Both *Sp* infection doses induced a shift in the MitoSOX fluorescence intensity compared to the medium control and the CSE-only condition (Figure 5a). Pre-treatment of cells with CSE for 24 h reduced the shift in the *Sp*-induced MitoSOX fluorescence signal, as compared to the *Sp* only infection. These results showed that *Sp* infection may increase mtROS levels in 16HBE cells, which was inhibited by prior exposure to CSE.

Secondly, the JC-1 test was performed to examine the impact of *Sp* infection alone and *Sp* infection in CSE pre-exposed cells on the MMP. Treatment with *Sp* alone (both MOI) initially resulted in increased MMP in 16HBE cells 1 h post-infection (24 h + 1 h; 25 h) and reduced MMP 4 h post-infection (24 h + 4 h; 28 h) in 16HBE cells (Figure 5b). However, pre-stimulation with CSE for 24 h followed by 4 h *Sp* infection (time point 28 h) reduced MMP to levels similar to the CSE-only condition (Figure 5b). These findings may show that *Sp* infection time-dependently affects MMP and that pre-exposure with CSE in combination with *Sp* may reduce MMP stronger than *Sp* infection alone. 

Next, immunoblotting was performed to assess the levels of key proteins involved in metabolic processes such as OXPHOS and glycolysis. Moreover, protein abundance of constituents of mitochondrial biogenesis were investigated upon CSE or *Sp* infection alone and with CSE pre-exposure in 16HBE cells (Figure 5c). The abundance of complex V (ATP5A) protein significantly decreased upon *Sp* infection with CSE pre-exposed cells compared to *Sp* only infection (Figure 5d). Moreover, *Sp* infections with CSE pre-stimulation significantly decreased the levels of complex II (SDHB) protein compared to the medium control (Figure 5e). *Sp* infection with CSE pre-exposure reduced glycolysis protein hexokinase II (HKII) compared to the medium control (Figure 5f). Importantly, the suppressing effects of CSE on HKII levels were inhibited by removing CSE from the medium after 4 h (CSE + Med) (Figure 5f), suggesting that the effect of CSE on HKII is transient. 

In addition, the levels of NRF1 clearly decreased after *Sp* infection in comparison to the medium control, while CSE pre-exposure had no additional effect on this (Figure 5g). Although CSE stimulation alone only tends to reduce the ERRα protein in 16HBE cells compared to the medium control (*p* = 0.05), we did observe that *Sp* infection with CSE pre-stimulation significantly reduced the abundance of ERRα only compared to *Sp* infection alone (Figure 5h), implying the strong additive effects on mitochondrial biogenesis when CSE pre-exposed cells were infected with *Sp*. Collectively, these results showed that *Sp* infection with CSE pre-exposure significantly diminishes mitochondrial processes in 16HBE cells.

Moreover, immunoblotting was carried out to probe selected factors that regulate mitochondrial fusion and fission (Figure 5i) as well as mitophagy (Figure 5l) upon CSE or *Sp* infection alone and with CSE pre-exposure. Although infection with *Sp* induced a significant decrease in the protein levels of MFN2 in 16HBE cells compared to the medium control, *Sp* infection with CSE pre-exposure did not affect the MFN2 protein (Figure 5j). Moreover, *Sp* infection with CSE pre-exposure did not alter the protein abundance of DRP1 compared to the medium control (Figure 5k), suggesting that *Sp* mainly affects mitochondrial fusion and CSE pre-exposure inhibits the effect of *Sp* infection. In addition, *Sp* infection with CSE pre-stimulation induced a significant decrease in abundance of BNIP3 compared to the medium control and *Sp* infection, and this effect on BNIP3 was less prominent than CSE alone, suggesting that *Sp* infection has no additive effect on the effects induced by CSE on BNIP3 (Figure 5m). Moreover, a significant decrease in the Fun14 domain-containing protein 1 (FUNDC1) protein expression was observed upon *Sp* infection with CSE pre-exposure compared to the *Sp* infection alone and to the medium control (Figure 5n), suggesting that *Sp* infection does not alter the effects induced by CSE pre-exposure on FUNDC1. The levels of PINK-I significantly enhanced upon infection with *Sp* with CSE pre-stimulation compared to the *Sp* infection only and the medium (Figure 5o), indicating that *Sp* infection has strong additive effect on PINK-I levels upon CSE pre-exposure. The abundance of GABARAPL1, SQSTM1, and LC3BII/I ratio increased upon *Sp* infection with CSE pre-exposure compared to the medium control (Figure 5p,q,r), showing that *Sp* infection has no additional effects on the effects induced by CSE pre-exposure on mitophagy adaptor protein levels. These data suggest that *Sp* infection with CSE pre-exposure may affect mitophagy stronger than CSE alone and in a different way than *Sp* infection alone.

In order to examine the ultrastructural changes induced by *Sp* infection alone and the impact of CSE on this, a transmission electron microscopy (TEM) analysis was carried out. Swollen mitochondria with damaged crista were clearly observed once untreated or CSE pre-exposed cells were infected with *Sp* only (MOI 10). The same aberrant mitochondrial morphology was observed for the cells treated with FCCP 50 µM (Figure S4).

Together, CSE affected 16HBE cells responses to *Sp* infection by altering the mitochondrial function and ultrastructure.

### 3.6. CSE Pre-Exposure Changes Expression Profiles of Genes Involved in Glycolysis, Innate Immune Responses, and Autophagy upon Sp Infection

**Figure 6:**
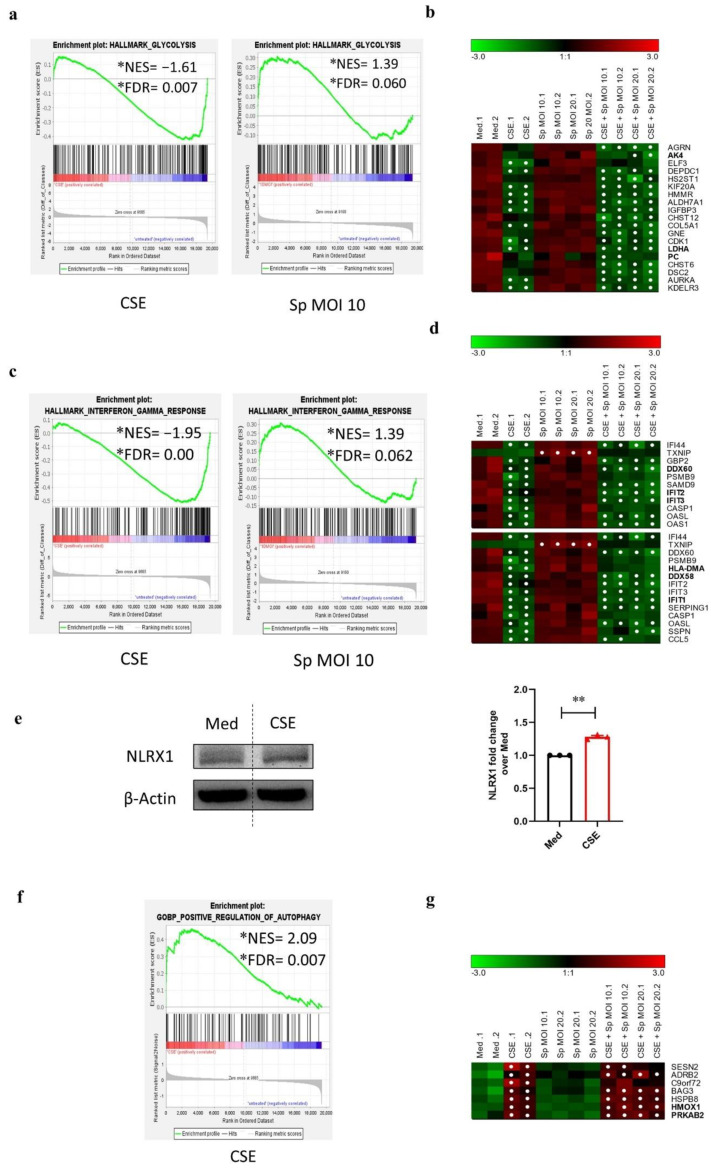
Gene expression and gene set enrichment analysis of 16HBE epithelial cell responses upon cigarette smoke extract stimulation and *Streptococcus pneumoniae* infection in 16HBE cells. Total-RNA from 16HBE cells from stated treatment conditions was isolated and transcriptome was analyzed with Clariom S Microarrays. For details on data analysis, see material and methods and text. Microarray data for each condition was analyzed by Gene Set Enrichment (GSEA) with untreated medium control as reference (ranked by fold change, FDR < 0.1). Normalized log_2_ signal intensities of core-enriched genes were z-score-transformed, color-coded, and plotted as heatmap (row labels: gene symbols). Details on bold gene symbols are mentioned in the text. Significance of differential expression with fold change cutoff |FC| > 3 in reference to the medium (Med) control was calculated by ANOVA (*p* < 0.05). White points in heatmap plots indicate |FC| > 3 with *p* < 0.05. Green/black/red color bars represent z-scores. Only selected significantly over-represented gene sets for (**a**) glycolysis, (**c**) interferon γ responses, and (**f**) positive regulation of autophagy with their according heat map of core-enriched genes (**b**,**d**,**g**) are shown. NES: normalized enrichment score, FDR: false discovery rate. (**e**) Immunoblotting of the NLR family member X1 (NLRX1) upon incubation of 16HBE cells with Med or CSE for 24 h (**left** panel). Normalized densitometrical quantification of NLRX1 protein expression (**right** panel). Bars and whiskers represent mean ± SEM (N = 3) of fold change over the medium control. Statistical significance was calculated by a paired *t*-test (** *p* < 0.001).

To explore the potential association between CSE-induced changes in mitochondrial gene expression and changes in expression of target genes belonging to epithelial cell responses to *Sp* infection in 16HBE cells, the expression of the relevant genes was analyzed using transcription microarray analysis of data. For details on microarray data analysis, see material and methods section. Briefly, heatmaps of differentially expressed genes (assessed by ANOVA; *p* < 0.05), with FC > 3 in at least one differential comparison, were z-score-transformed and k-means-clustered (Figure S5). The result was a combined Gene set enrichment analysis (GSEA) of hallmark sets, selected Gene-Ontology-based gene sets, and selected gene sets provided by the Reactome database. GSEA was calculated for each condition with fold-change-based ranking, using the untreated condition as universal reference. Significantly overrepresented gene sets (FDR < 0.1) were restricted to GSEA-core-enriched genes, whose expression data were then z-score-transformed, clustered, plotted as heatmap, and combined with the results of the initial differential ANOVA analysis, but showed a total of 834 differentially regulated genes over all the condition comparison. GSEA analysis showed that the genes encoding for proteins belonging to the mitochondrial ribosomal translation and import machinery are enriched upon infection with *Sp* (MOI 10) alone and with CSE pre-exposure compared to the medium control (NES: 2.02 and 1.91, respectively) (Figure S6 and Figure 6b), implying that *Sp* has more distinct effect on mitochondrial gene expression than CSE.

Interestingly, *Sp* infection with CSE pre-stimulation and CSE stimulation alone decreased the normalized enrichment score of the genes involved in glycolysis (Figure 6a) and several of these genes were significantly downregulated compared to the medium control (Figure 6b). In contrast, the expression of these genes was high in both, the medium control and *Sp* infection alone, but became downregulated in all other conditions as soon as CSE was involved (Figure 6b).

Moreover, data from GSEA analysis showed that *Sp* infection alone drives functional enrichment of the genes involved in complement (NES: 1.23), interferon α (NES: 1.29), and interferon γ responses (NES: 1.39). Expression of the according genes within those gene sets as well as the major histocompatibility complex (MHC) class II pathway was significantly reduced upon *Sp* infection with CSE pre-exposure and CSE alone (Figure S7 and Figure 6c). This is in line with significant downregulation of interferon γ response genes in CSE stimulated conditions (Figure 6d). Moreover, CSE significantly downregulated *c-c motif chemokine 5* (*CCL5*) and *Toll-like receptor 3* (*TLR3*) while these genes were upregulated upon *Sp* only infection (cluster 1 and 5, Figure S5). In order to find the mechanistic factor linking the observed decrease in interferon responses to mitochondria, we investigated the abundance of the mitochondrial-located NOD-like receptor X1 (NLRX1) as a mitochondrial factor known to suppress interferon responses [15]. We observed that CSE stimulation of 16HBE cells for 24 h resulted in significantly increased abundance of the NLRX1 protein (Figure 6e), suggesting a potential contribution of mitochondrial damage to diminished interferon responses upon CSE.

Furthermore, autophagy-related genes were significantly GSE-enriched (NES: 2.09) in CSE only stimulation conditions compared to the untreated medium (Figure 6f). Genes in this category became however upregulated in all three CSE-treated conditions, including CSE pre-exposure with *Sp* infection. Moreover, expression of SQSTM1, which was observed to be increased at protein level, was upregulated in all CSE-treated conditions (cluster 4, Figure S5). Contrary, expression of autophagy genes remained relatively unchanged upon *Sp* infection alone (Figure 6g).

Collectively, these data show that while *Sp* infection alone induced the expression of genes related to innate immune responses, CSE stimulation decreased these responses, in particular interferon responses and cell surface antigen recognition in 16HBE cells. These CSE-induced changes in genes regulating innate immune responses were accompanied with an increased abundance of the negative regulator of interferon responses NLRX1.

## 4. Discussion

In this study, we investigated the impact of acute exposure of 16HBE cells to CSE on the regulation of mitochondrial function in the presence or absence of subsequent infection with *Sp*. The major aim was to investigate the impact of mitochondrial abnormalities (induced by CSE) on airway epithelial responses, particularly innate immune responses, and the airway epithelial barrier function. Moreover, we sought to determine mitochondrial changes and innate immune responses in 16HBE cells by CSE and *Sp* alone and in combination.

We have demonstrated that short-term CSE exposure induced mitochondrial dysfunction in AECs by enhancing the mitochondrial oxidative stress, loss of MMP as well as by reducing mitochondrial complex protein levels, which is in line with previous studies showing similar mitochondrial dysfunction in AECs upon short and long-term exposure to low doses of CSE [11,16]. In addition, CSE affected the regulation of mitochondrial quality control processes by attenuating mitochondrial fusion-associated markers and altering the abundance of constituents associated with mitochondrial fission and mitophagy. The abnormal mitophagy was identified through increased mitochondrial protein levels of LC3B and SQSTM1 as well as decreased receptor-mediated mitophagy constituents, respectively BNIP3 and FUNDC1, in AECs upon CSE exposure. Several mechanistic explanations can be suggested for the observed imbalanced abundance of mitophagy-associated constituents upon CSE, respectively regulation via mTOR signaling, oxidative stress response or AMPK regulation. 

Firstly, the LC3B-SQSTM1-mediated increase in mitophagy may not be regulated by mTOR signaling, the negative regulator of autophagy, as only a subtle upregulation of genes involved in the mTORC1 pathway, particularly SQSTM1 and CDKN1a were observed upon CSE. Interestingly, CSE deprivation from the medium was able to counteract the enrichment in mTORC1 gene set, indicating that the activation of mTOR may be transient (data not shown). Secondly, the influence of an increased oxidative stress response in CSE-induced mitophagy was ruled out by using the mtROS inducer FCCP, which did not alter the levels of mitophagy markers. Thirdly, we hypothesized that AMPK may regulate GABARAPL1-mediated mitophagy upon CSE stimulation, as it is known that AMPKα phosphorylation positively regulates initiation of autophagy via phosphorylation of ULK1 at serine 555 [17]. AMPKα is a metabolic sensor which is dependent on mitochondrial OXPHOS activity, and a decrease in its phosphorylated form has been reported in the lung of COPD patients [18]. It is postulated that the levels of phosphorylated AMPKα initially increases in response to CSE to resolve the inflammation by increasing antioxidant regulators such as mitochondrial superoxide dismutase 2 (SOD2) and nuclear factor erythroid 2-like 2 (NRF2) [18,19,20]. However, it is incompletely understood whether this pattern of AMPKα phosphorylation is associated with the severity of the stage of COPD. In contrast, several studies reported that CSE exposure resulted in decreased AMPKα phosphorylation in AECs in a time-dependent manner both in vitro and in vivo [21,22]. In our study, we have observed that stimulation of AECs with CSE resulted in slightly reduced phosphorylation of AMPKα at Thr172, while it upregulated AMPKβ2 gene expression, suggesting a potential involvement of other subunits of AMPK in CSE-induced increase in mitophagy. Phosphorylation of ULK1 by AMPK leads to its translocation to the mitochondria and an increased abundance of constituents involved in the mitophagy machinery [23]. Immunoblotting for phospho-ULK1 at Ser555 only showed a slight increase upon stimulation with CSE for 14 h, suggesting that the increased mitophagy induced by CSE was minimally affected by the AMPKα/ULK1 pathway and mainly triggered by other mitophagy regulators.

Besides our finding on CSE-induced impairment in mitochondrial function and disrupted regulation of mitochondrial quality control processes in AEC, the existing literature has shown that CS exposure dysregulates innate immune responses to pathogens resulting in a secondary bacterial infection with, e.g., *Sp* serotypes [8,24,25]. *Sp* serotypes are mainly composed of non-invasive and invasive strains [26], of which non-invasive common nasal colonizer serotypes such as serotype 15A, 19A, 19F, and 23B were frequently isolated from patients with airway diseases [27]. *Sp* serotype 19F is one of the increasingly isolated serotypes that does not respond to routine antimicrobial treatments [28]. It was reported that *Sp* infection increases the oxidative burden and reduces MMP in the lung of aged mice [29]. Furthermore, stimulation with virulence factors of *Sp* pneumolysin and hydrogen peroxide were shown to induce mitochondrial membrane permeability in alveolar epithelial cells leading to mitochondrial DNA release and a pro-inflammatory response [30,31]. We have shown that while infecting 16HBE cells with *Sp* increased mtROS levels, this *Sp* treatment did not alter protein levels of subunits of mitochondrial OXPHOS complexes, suggesting that changes in mitochondrial components are not necessarily ROS-dependent upon *Sp* infection in AECs. Furthermore, *Sp* infection enhanced the abundance of mitochondrial fission protein DRP1, showing that increased mitochondrial fragmentation may proceed with the cell death in *Sp* infection. This is also in line with the observed decrease in abundance of constituents controlling mitochondrial biogenesis and abnormal mitochondrial morphology, respectively loss of crista and mitochondrial swelling in response to *Sp*. Moreover, increased hypoxia-mediated mitophagy levels as well as increased levels of HKII, responsible for the first step in glycolysis, and enhanced enrichment of genes involved in glycolysis were observed upon *Sp* infection. In contrast, prior CSE exposure followed by *Sp* infection mainly induced similar but stronger changes than CSE alone on the abundance of mitochondrial proteins involved in mitophagy and OXPHOS/glycolysis in 16HBE cells, suggesting potential changes in mitophagy and OXPHOS/glycolysis processes. Together, infection with non-invasive *Sp* alone elicited different mitochondrial abnormalities than those induced by CSE alone, and *Sp* infection with CSE pre-exposure exacerbated the mitochondrial dysfunction in 16HBE cells. 

Inhaled pathogens and noxious gases first encounter physical and chemical barriers in AECs as the gatekeeper of innate immune responses [2]. The mitochondrial function may regulate the innate immune response during infection and inflammation [3]. Although early pro-inflammatory responses to the pathogens may resolve the infection and mitigate the damage, chronic infection with persistent inflammation may break this barrier [32]. We observed that CSE exposure induced pro-inflammatory responses by increasing acute phase response gene expression of cytokines, for example, neutrophil attractants, such as *CXCL8* and *IL-23A*, and *IL1β*. This early protective inflammatory response was confirmed in our model by the observation that pre-incubation of 16HBE cells with CSE reduces the bacterial viability (data not shown). Contrary, CS is known to suppress certain antimicrobial responses of airway epithelium to pathogens such as epithelial β-defensins [33]. We observed that while *Sp* infection increased type I and II interferon responses as well as complement activity at mRNA levels, CSE exposure dampened these responses in AECs. In line, several studies reported that CS exposure suppresses innate immune responses in AECs by negatively regulating IFN responses [34,35,36]. The CSE-induced impairment in IFN responses and consequent dampened innate immune suppression may be triggered through downregulation of RIG-I, MDA-5 and TLR3 via IKB kinase epsilon (IKBKE)-mediated regulation of interferon regulatory factors (IRFs), as observed by downregulation of *RIG-I*, *MDA-5*, and *TLR3* genes upon CSE stimulation. Additionally, CSE-induced attenuation of interferon responses may be triggered by increased levels of mitochondrial antiviral signaling (MAVS) inhibitors such as E3 ligases Trim29, Smurf1/2, March5 that degrade MAVS or by NLRX1-mediated MAVS inhibition [37]. This NLRX-mediated inhibition of MAVS and downregulation in interferon response is reported to be regulated via the Tu mitochondrial translation elongation factor (TUFM) [38]. In addition to the effects on interferon responses, mitochondrially localized NLRX1 has been reported to affect mitophagy via TUFM [38] or via direct interaction with LC3B [39]. We have observed that CSE exposure enhances NLRX1 protein levels, which may subsequently inhibit MAVS and lead to the observed dampened interferon responses and likely contributes to the increased mitophagy. Therefore, our findings suggest that CSE-induced mitochondrial dysfunction may contribute to impaired interferon responses and increased mitophagy especially at the earlier phase of infection.

Next, we used mitochondrial-targeted compounds to untangle the impacts of improving mitochondrial function on restoration of CSE-induced disrupted airway epithelial physical barriers. We found that post-treatment of AECs with both mitochondrial-derived peptide MOTS-c as well as mitochondrial antioxidant MitoTEMPO restores tight and adherens junctions disrupted by CSE exposure. MOTS-c is a mitochondrial short open reading frame peptide with a potential metabolic activity by activating AMPK and increasing the glucose uptake [40]. Furthermore, MOTS-c improves sirtuin 1 through increasing NAD+ levels and thus enhances glycolysis [40]. To our knowledge, this is the first report showing an epithelial barrier protective effect for MOTS-c. The barrier protective effects of both mitochondrial compounds may be triggered via improvement of mitochondrial biogenesis via increased glycolysis as already reported for MitoTEMPO in AECs upon rhinovirus infection [41]. 

Together, CSE-induced mitochondrial dysfunction may contribute to a weakened immunological and physical epithelial barrier function in AECs. Our study was, however, limited by several technical issues. Firstly, we only used a monolayer of undifferentiated and immortalized airway epithelial cells. In physiological condition, AECs function in interaction with other airway structural cells as well as resident and circulated immune cells [42]. Moreover, CSE that has been used in this study mainly contains soluble gas phase contents, excluding the particle phase. Finally, mitochondrial function such as respiration or acidification could be assessed by more functional assays. Future studies will further illuminate the impacts of mitochondrial dysfunction induced by exposing animals or differentiated AECs to whole CS on the epithelial responses by considering the interaction with resident and circulating immune cells.

## Data Availability

Microarray data were deposited in National Center for Biotechnology Information’s (NCBI’s) Gene Expression Omnibus (GEO) and are accessible through the GEO series accession number GSE197751 (https://www.ncbi.nlm.nih.gov/geo/query/acc.cgi?acc=GSE197751; accessed on 23 May 2022).

## Supplementary Materials

The following supporting information can be downloaded at: https://www.mdpi.com/article/10.3390/cells11111771/s1, Figure S1: Growth curve of Streptococcus pneumoniae upon CSE exposure. Sp growth curve in presence and absence of different concentrations of cigarette smoke extract (CSE) (5–25%) for 5 h. Data were represented as mean ± SEM of triplicate samples for each condition.; Figure S2: Optimization of CSE-induced mitochondrial dysfunction model. 16HBE cells were incubated with medium control or various concentrations of cigarette smoke extract (CSE) (5, 10, 15 and 20%) for 24 h. (a) Proliferation of the treated 16HBE cells was quantified using 3-(4,5-dimethylthiazole-2-yl) 2,5-diphenyl-2H-tetrazolium bromide (MTT) test. The data is presented as mean of triplicates (±SEM) and representative of one out of two independent experiments with similar outcomes. (b) Real-Time Glo MT assay for time-point measurement of cell proliferation until time 19 h. The data is representative of one out of two independent experiments with similar outcome in triplicates. (c) Total reactive oxygen species (ROS) levels analyzed with dichlordihydrofluorescein diacetate (H2-DCFDA) test presented as mean fluorescent intensity (MFI) of triplicates (±SEM) normalized to measurement at assay start (0 h). Tert-butyl hydroperoxide (tBHP) 50 µM was used as positive regulator of ROS. Statistically significant difference was calculated with multiple t-test compared to the medium control (* *p* < 0.05). The data is representative of one out of three independent experiments with similar outcome and in triplicates. (d) Half offset histogram was used to display shift in the MitoSOX fluorescent signal detected by FACS and analyzed by FlowJo, indicative of changes in mitochondrial ROS levels (left panel) and fold change of MFI MitoSOX upon CSE and carbonyl cyanide-p-trifluoromethoxyphenylhydrazone (FCCP; positive regulator of mitochondrial ROS). MitoSOX MFI fold change was calculated for each condition compared to the medium. The MFI is representative of two independent experiments. (e) Mitochondrial membrane potential was measured using JC-1 test by dividing J-aggregate (red signal) by monomer (green signal) and normalized to the ratio in the corresponding condition at 0 h. The data is representative of one experiment out of two independent experiments with triplicate samples; Figure S3: Immunostaining for estrogen related receptor α (ERRα). The 16HBE cells were stimulated with medium control, cigarette smoke extract (CSE) for 28 h, CSE 24 h followed by mitochondrial ORF of the 12S rRNA type-c (MOTS-c) 25 µM for 4 h or with carbonyl cyanide-p- trifluoromethoxyphenylhydrazone (FCCP) 50 µM. Immunofluorescence staining was performed using an antibody against ERRα (red) and 4′,6-diamidino-2-phenylindole (DAPI) (blue) for nuclei staining. The scale bar is equivalent to 20 µm. Figure S4: Ultrastructure of 16HBE cells upon CSE stimulation and *Streptococcus pneumoniae*. Mitochondrial morphological changes were analyzed under transmission electron microscope (TEM). Dark arrows indicating normal mitochondrial morphology, whereas red arrows showing loss of crista and swollen mitochondria. Representative images of 16HBE cells incubated with (a) medium for 28 h, (b) cigarette smoke extract (CSE) for 28 h, (c) *Streptococcus pneumoniae* (*Sp*) multiplicity of infection (MOI) 10 for 4 h, (d) CSE for 24 h followed by Sp MOI 10 for 4 h (e), CSE for 24 h followed by medium for 4 h and (f) carbonyl cyanide-p-trifluoromethoxyphenylhydrazone (FCCP) 50 µM for 4 h. The scale bar is equivalent to 2 µm; Figure S5: Microarray analysis of gene expression upon CSE stimulation and *Streptococcus pneumoniae* infection. 16HBE cells were stimulated with medium (Med) or cigarette smoke extract (CSE) for 28 h, CSE for 24 h followed by medium 4 h (CSE cessation), *Streptococcus pneumoniae* (*Sp*) multiplicities of infection (MOI) of 10 and 20 for 4 h, CSE for 24 h followed by *Sp* MOI 10 and 20 for 4 h. Total RNA was isolated and investigated by using human Clariom S microarray. Differentially expressed transcripts were determined by comparing each condition with medium control (fold change ≥3-fold, false discovery rate <0.05). Log_2_ signal intensities data of the transcripts were z-score transformed and k-means clustered (k = 8), and transcripts in resulting clusters were sorted by maximal absolute z-score (row labels: gene symbols). Data represent color-coded z-scores. Details on bold gene symbols are mentioned in the text. Figure S6: Expression of genes regulating mitochondrial function in 16HBE cells upon *Streptococcus pneumoniae* infection with and without CSE pre-exposure. Gene set enrichment analysis (GSEA) for mitochondrial ribosomal translation and mitochondrial import Reactome genes upon (a) infecting 16HBE cells with *Streptococcus pneumoniae* (*Sp*) multiplicity of infection (MOI) 10 for 4 h and (b) incubation with cigarette smoke extract (CSE) for 24 h followed by *Sp* infection with MOI 10 for 4 h compared to the untreated medium (ranked by fold change, FDR < 0.1). The data were collected from two independent experiments; Figure S7: Expression of genes regulating innate immune responses upon cigarette smoke extract stimulation and *Streptococcus pneumoniae* infection. (a) Gene set enrichment analysis (GSEA) for cell surface major histocompatibility complex II (MHCII) in Reactome gene set upon cigarette smoke extract (CSE) stimulation compared to the medium (Med) control. Normalized log_2_ signal intensities of core-enriched genes were z-score transformed, color-coded and plotted as heatmap (row labels: gene symbols). Details on bold gene symbols are mentioned in the text. Significance of differential expression with fold change cut-off |FC| > 3 in reference to Med control was calculated by ANOVA (*p* < 0.05). White points in heatmap plots indicate |FC | > 3 with *p* < 0.05. Green/black/red color bars represent z-scores. (b) GSEA for interferon α and γ hallmarks upon infection of 16HBE cells with multiplicity of infection (MOI) 10 of *Streptococcus pneumoniae* (*Sp*). (c) GSEA for complement hallmarks upon stimulation of 16HBE cells with CSE for 28 h and *Sp* MOI 20 for 4 h compared to the untreated medium as well as regulated genes in this hallmark calculated by ANOVA (*p* < 0.05). The data were collected from two independent experiments. * NES: Normalized enrichment score, FDR: false discovery rate; Figure S8: Airway epithelial barrier disruption induced by cigarette smoke extract is reversible with mitochondrial-targeted compounds. 16HBE cells incubated for 28 h with medium (control) or cigarette smoke extract (CSE) for 28 h, or for 24 h with CSE followed by incubation for further 4 h with either mitochondrial-targeted compounds MOTS-c (25 µM) or MitoTEMPO (50 µM). (a) Immunofluorescence imaging of treated cells co-stained with antibodies against translocase of the outer mitochondrial membrane complex subunit 20 (TOMM20) in red, tight junction protein zonula occludens (ZO-1) in green or the nucleus with 4′,6-diamidino-2-phenylindole (DAPI) for nucleus staining (blue). (b) Treated cells were (co-)stained with anti-TOMM20-antibody (red) and anti-E-cadherin-antibody (green) or with nuclear staining dye DAPI (blue). False color-coded image columns shown here to depict differences in intensities. To visualize the barrier disruption, magnified regions of interest are indicated in the fourth column and shown in column five. Merged images have been gamma corrected to visualize weak signals without losing the highlights. The scale bar is equivalent to 20 µm (a) and 10 µM (b); Figure S9: Quantification of signal intensities of immunofluorescence staining in 16HBE cells. The cells were stained with antibodies against (a) dynamin-related protein 1 (DRP1) and (b) translocase of the outer mitochondrial membrane complex subunit 20 (TOMM20). Mean fluorescence intensity (MFI) was calculated for each cell in the corresponding immunofluorescence images by Fiji software. The statistically significant differences with the medium (Med) were determined by unpaired t-test and using Welch’s post-hoc test (** *p* < 0.001, *** *p* < 0.0001); Table S1: Antibodies used for immunoblotting. Abbreviations: AMPKα, AMP-activated protein kinase; p-AMPKα, Phosphorylated AMP-activated protein kinase; β-Actin, beta-Actin; BNIP3, BCL2-interacting protein 3; BNIP3L, BCL2 interacting protein 3-like; COXIV, Cytochrome c oxidase subunit IV; DRP1, Dynamin-related protein 1; ERRα, Estrogen-related receptor alpha; FUNDC1, Fun14 domain-containing protein 1; GABARAPL1, Gamma-aminobutyric acid receptor-associated protein-like 1; GAPDH, Glyceraldehyde-3-phosphate dehydrogenase; HKII, hexokinase II; HRP, Horseradish peroxidase; LC3B, Microtubule-associated protein 1A/1B-light chain 3B; MFN2, Mitofusion 2; NLRX1, Nucleotide-binding domain and leucine-rich repeat–containing protein X1; NRF1, Nuclear respiratory factor 1; OXPHOS, Oxidative phosphorylation antibody cocktail (containing SDHB: Succinate dehydrogenase subunit B, UQCRC2: Ubiquinol-cytochrome C reductase core protein 2, ATP5A: adenosine triphosphate 5A); PINK, Phosphatase and tensin homolog (PTEN)-induced kinase; SQSTM1, Sequestosome 1; TOMM20, Translocase of the outer mitochondrial membrane complex subunit 20; ULK1, Unc-51 like autophagy activating kinase 1; p-ULK1, Phosphorylated Unc-51 like autophagy activating kinase 1; VDAC1, Voltage-dependent anion channel 1; Table S2: Antibodies used for immunocytochemistry. Abbreviations: DRP1, Dynamin-related protein 1; p-DRP1, phosphorylated dynamin-related protein 1; ERRα, Estrogen-related receptor alpha; TOMM20, Translocase of the outer mitochondrial membrane complex subunit 20; ZO-1, Zonula occludens 1.
