# Supplementary material for: Cigarette Smoke Extract Disturbs Mitochondria-Regulated Airway Epithelial Cell Responses to Pneumococci
Source: Cells. 2022 May 28;11(11):1771. doi: 10.3390/cells11111771 (PMC9179351; doi:10.3390/cells11111771)
Supplement: Supplementary file 1 [file cells-11-01771-s001.zip › cells-1728933-supplementary.pdf]

Supplementary Information

Cigarette smoke extract disturbs mitochondria-regulated airway epithelial cell responses to pneumococci

Mahyar Aghapour[1,2], Christy B.M. Tulen[3], Mohsen Abdi Sarabi[4], Sönke Weinert[4], Mathias Müsken[5], Borna Relja[6], Frederik-Jan van Schooten[3], Andreas Jeron[1,2], Rüdiger Braun-Dullaeus[4], Alexander H Remels[3], Dunja Bruder[1,2]

[1]Infection Immunology Group, Institute of Medical Microbiology, Infection Control and Prevention, Health Campus Immunology, Infectiology and Inflammation, Otto-von-Guericke University, Magdeburg, Germany

[2]Immune Regulation Group, Helmholtz Centre for Infection Research, Braunschweig, Germany

[3]Department of Pharmacology and Toxicology, School of Nutrition and Translational Research in Metabolism (NUTRIM), Maastricht University Medical Center +, Maastricht, The Netherlands

[4]Department of Internal Medicine/Cardiology and Angiology, Otto-von-Guericke University, Magdeburg, Germany

[5]Central Facility for Microscopy, Helmholtz Centre for Infection Research, Braunschweig, Germany

[6]Experimental Radiology, Department of Radiology and Nuclear Medicine, Magdeburg, Germany

Supplemental Tables

Antibody	Company	Working concentrations	Cat-number	Clone
**Primary antibodies**				
AMPK-α	Cell signaling Technology	1:1000	2532	-
p-AMPKα (Thr172)	Cell signaling Technology	1:1000	2535	40H9
β-Actin	Cell signaling Technology	1:2000-1:5000	4970	13E5
BNIP3	Cell signaling Technology	1:1000	3769S	-
BNIP3L	Cell signaling Technology	1:1000	12396	D4R4B
COXIV	Cell signaling Technology	1:1000	4850	3E11
DRP1	Cell signaling Technology	1:1000	8570	D7C7
ERRα	Abcam	1:1000	ab76228	EPR46Y
FUNDC1	Santa Cruz Biotechnology	1:500	sc-133597	H22
GABARAPL1	Proteintech Group	1:1000	11010-1-AP	-
GAPDH	Cell signaling Technology	1:5000-1:10000	5174	D16H11
HKII	Cell signaling Technology	1:1000	2867	C64G5
LC3B	Cell signaling Technology	1:1000	2775	-
MFN2	Santa Cruz Biotechnology	1:500	sc-515647	F-5
NLRX1	Cell signaling Technology	1:1000	13829	D4M3Z
NRF1	Abcam	1:1000	ab55744	2F9
OXPHOS subunits	MitoScience LLC	1:1000	MS604	-
PINK	Novus Biologicals	1:2000	BC100-494	-
SQSTM1 (p62)	Cell signaling Technology	1:1000	5114	-
TOMM20	Abcam	1:1000	ab186734	EPR15581-39
ULK1	Cell signaling Technology	1:1000	8054	D8H5
p-ULK1 (Ser555)	Cell signaling Technology	1:1000	5869	D1H4
VDAC1	Cell signaling Technology	1:500	4866	-
**Secondary antibodies**				
Polyclonal Goat anti-Mouse IgG HRP	Vector laboratories	1:10000	BA-9200	-
Polyclonal Goat anti-Rabbit IgG HRP	Cell signaling Technology	1:10000	7074S	-
Polyclonal Swine anti-	Dako-Agilent	1:1000-1:10000	P0399	-

Rabbit IgG HRP				
Polyclonal Rabbit anti-Mouse IgG HRP	Dako-Agilent	1:1000-1:10000	P0161	-

Table S1: Antibodies used for immunoblotting. Abbreviations: AMPKα, AMP-activated protein kinase; p-AMPKα, Phosphorylated AMP-activated protein kinase; β-Actin, beta-Actin; BNIP3, BCL2-interacting protein 3; BNIP3L, BCL2 interacting protein 3-like; COXIV, Cytochrome c oxidase subunit IV; DRP1, Dynamin-related protein 1; ERRα, Estrogen-related receptor alpha; FUNDC1, Fun14 domain-containing protein 1; GABARAPL1, Gamma-aminobutyric acid receptor-associated protein-like 1; GAPDH, Glyceraldehyde-3-phosphate dehydrogenase; HKII, hexokinase II; HRP, Horseradish peroxidase; LC3B, Microtubule-associated protein 1A/1B-light chain 3B; MFN2, Mitofusion 2; NLRX1, Nucleotide-binding domain and leucine-rich repeat–containing protein X1; NRF1, Nuclear respiratory factor 1; OXPHOS, Oxidative phosphorylation antibody cocktail (containing SDHB: Succinate dehydrogenase subunit B, UQCRC2: Ubiquinol-cytochrome C reductase core protein 2, ATP5A: adenosine triphosphate 5A); PINK, Phosphatase and tensin homolog (PTEN)-induced kinase; SQSTM1, Sequestosome 1; TOMM20, Translocase of the outer mitochondrial membrane complex subunit 20; ULK1, Unc-51 like autophagy activating kinase 1; p-ULK1, Phosphorylated Unc-51 like autophagy activating kinase 1; VDAC1, Voltage-dependent anion channel 1.

Antibody	Company	Working concentration	Cat-number	Clone
**Primary antibodies**				
DRP1	Santa Cruz Biotechnology	1:50	sc-271583	C-5
p-DRP1 (Ser616)	Cell signaling	1:300	3455	-
TOMM20 (F-10)	Santa Cruz Biotechnology	1:50	sc-17764	-
ZO-1	Cell signaling	1:400	13663	D6L1E
E-cadherin	Cell signaling	1:200	3195	24E10
ERRα	Abcam	1:100	EPR46Y	ab76228
**Secondary antibody**				
Anti-rabbit IgG Fab2 Alexa fluor (R) 488	Cell signaling	1:1000	4412S	-
Anti-rabbit IgG Fab2 Alexa fluor (R) 647	Cell signaling	1:1000	4410S	-
Alexa fluor 647 Donkey Anti-Rabbit IgG	Biolegend	1:1000	406414	Poly4064

Table S2: Antibodies used for immunocytochemistry. Abbreviations: DRP1, Dynamin-related protein 1; p-DRP1, phosphorylated dynamin-related protein 1; ERRα, Estrogen-related receptor alpha; TOMM20, Translocase of the outer mitochondrial membrane complex subunit 20; ZO-1, Zonula occludens 1.

Supplemental Figures and Figures and Legends

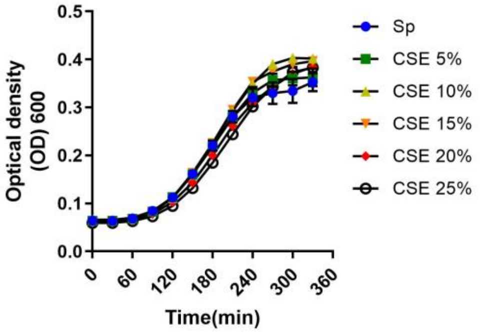

Figure S1: Growth curve of *Streptococcus pneumoniae* upon CSE exposure. *Sp* growth curve in presence and absence of different concentrations of cigarette smoke extract (CSE) (5-25%) for 5 h. Data were represented as mean ± SEM of triplicate samples for each condition.

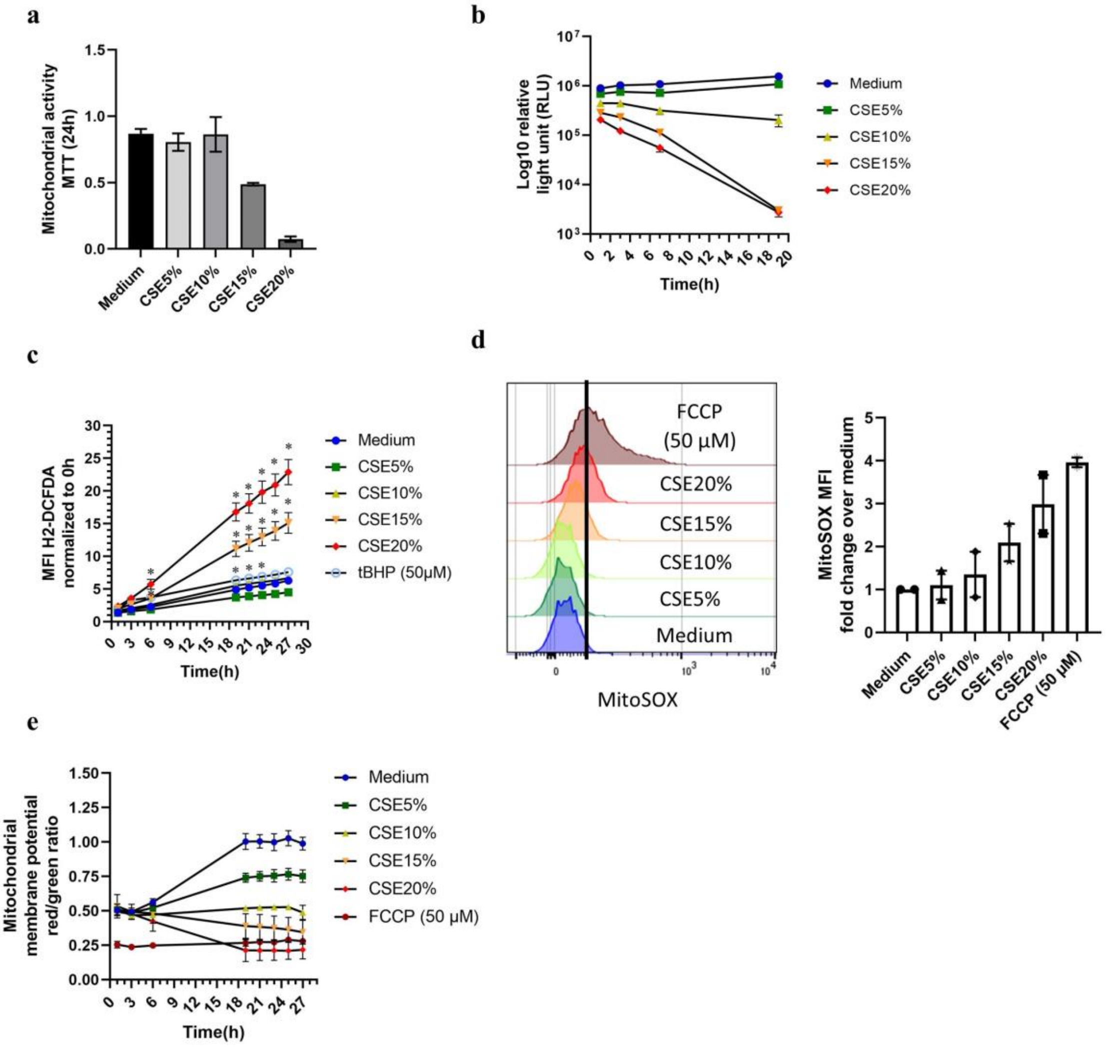

Figure S2: Optimization of CSE-induced mitochondrial dysfunction model. 16HBE cells were incubated with medium control or various concentrations of cigarette smoke extract (CSE) (5, 10, 15 and 20%) for 24 h. **a)** Proliferation of the treated 16HBE cells was quantified using 3-(4,5-dimethylthiazole-2-yl) 2,5-diphenyl-2H-tetrazolium bromide (MTT) test. The data is presented as mean of triplicates (± SEM) and representative of one out of two independent experiments with similar outcomes. **b)** Real-Time Glo MT assay for time-point measurement of cell proliferation until time 19 h. The data is representative of one out of two independent experiments with similar outcome in triplicates. **c)** Total reactive oxygen species (ROS) levels analyzed with dichlordihydrofluorescein diacetate (H2- DCFDA) test presented as mean fluorescent intensity (MFI) of triplicates (± SEM) normalized to measurement at assay start (0 h). Tert-butyl hydroperoxide (tBHP) 50 µM was used as positive regulator of ROS. Statistically significant difference was calculated with multiple t-test compared to the medium control (*$p<0.05$). The data is representative of one out of three independent experiments with similar outcome and in triplicates. **d)** Half offset histogram was used to display shift in the MitoSOX fluorescent signal detected by FACS and analyzed by FlowJo, indicative of changes in mitochondrial ROS levels (left panel) and fold change of MFI MitoSOX upon CSE and carbonyl cyanide-p-trifluoromethoxyphenylhydrazone (FCCP; positive regulator of mitochondrial ROS). MitoSOX MFI fold change was calculated for each condition compared to the medium. The MFI is representative of two independent experiments. **E)** Mitochondrial membrane potential was measured using JC-1 test by dividing J-aggregate (red signal) by monomer (green signal) and normalized to the ratio in the corresponding condition at 0 h. The data is representative of one experiment out of two independent experiments with triplicate samples.

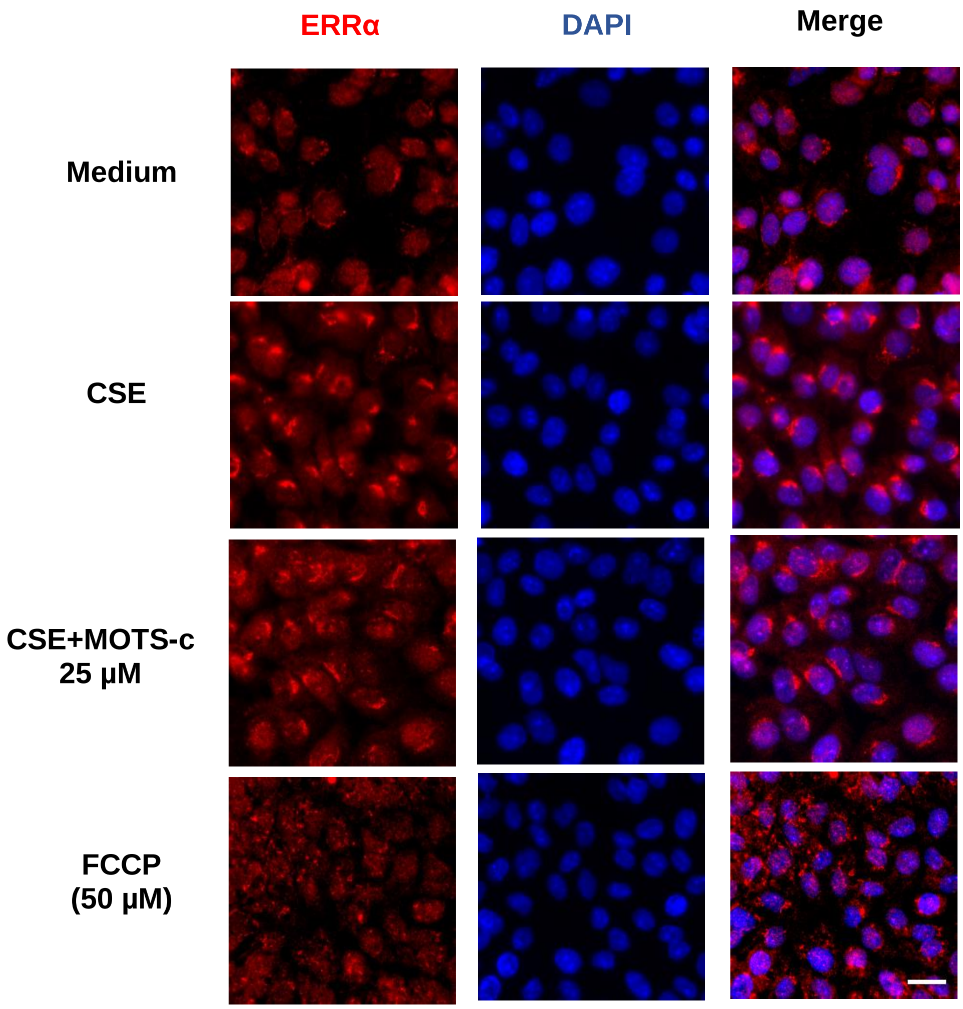

Figure S3: Immunostaining for estrogen related receptor α (ERRα). The 16HBE cells were stimulated with medium control, cigarette smoke extract (CSE) for 28 h, CSE 24 h followed by mitochondrial ORF of the 12S rRNA type-c (MOTS-c) 25 µM for 4 h or with carbonyl cyanide-p-trifluoromethoxyphenylhydrazone (FCCP) 50 µM. Immunofluorescence staining was performed using an antibody against ERRα (red) and 4′,6-diamidino-2-phenylindole (DAPI) (blue) for nuclei staining. The scale bar is equivalent to 20 µm.

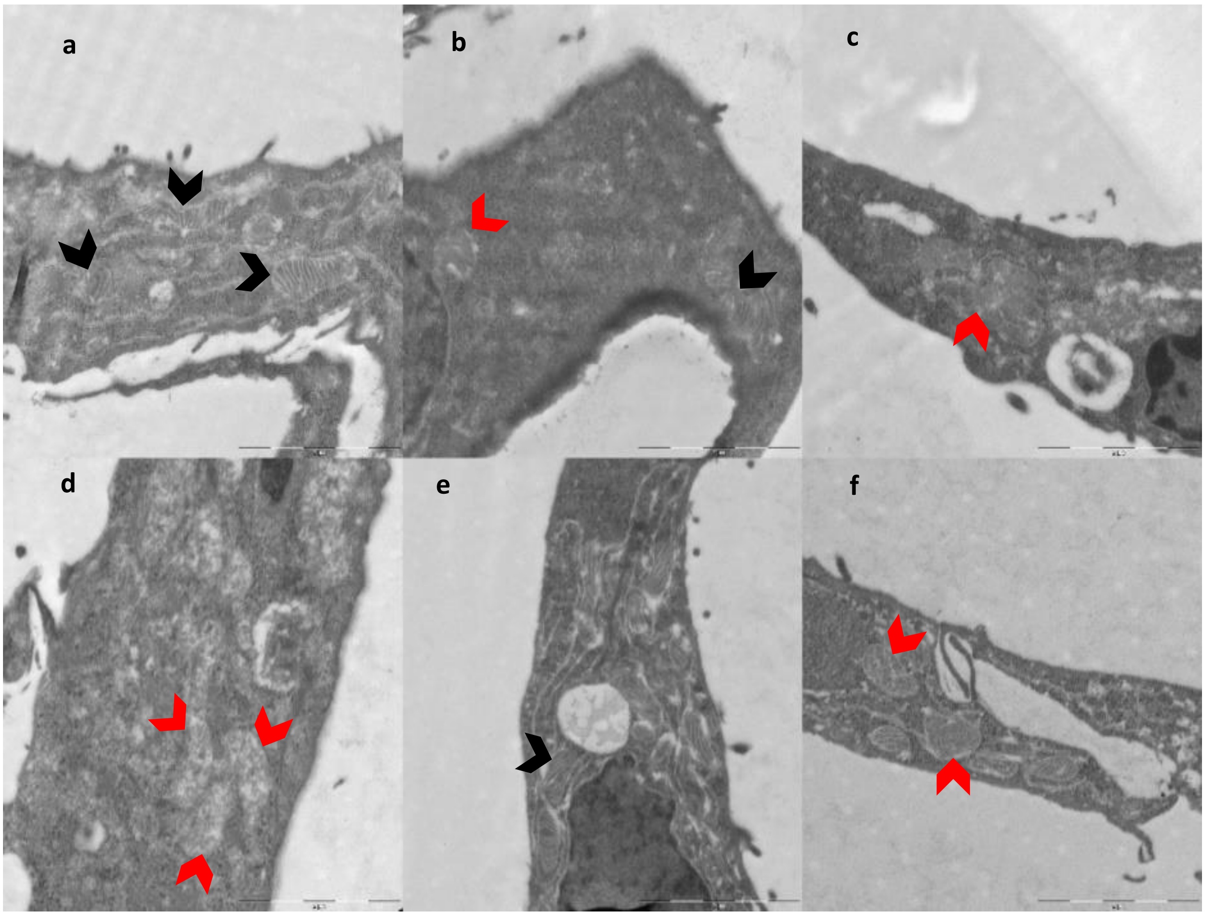

Figure S4: Ultrastructure of 16HBE cells upon CSE stimulation and *Streptococcus pneumoniae*. Mitochondrial morphological changes were analyzed under transmission electron microscope (TEM). Dark arrows indicating normal mitochondrial morphology, whereas red arrows showing loss of crista and swollen mitochondria. Representative images of 16HBE cells incubated with **a)** medium for 28 h, **b)** cigarette smoke extract (CSE) for 28 h, **c)** *Streptococcus pneumoniae* (*Sp*) multiplicity of infection (MOI) 10 for 4 h, **d)** CSE for 24 h followed by *Sp* MOI 10 for 4 h **e)**, CSE for 24 h followed by medium for 4 h and **f)** carbonyl cyanide-p-trifluoromethoxyphenylhydrazone (FCCP) 50 µM for 4 h. The scale bar is equivalent to 2 µm.

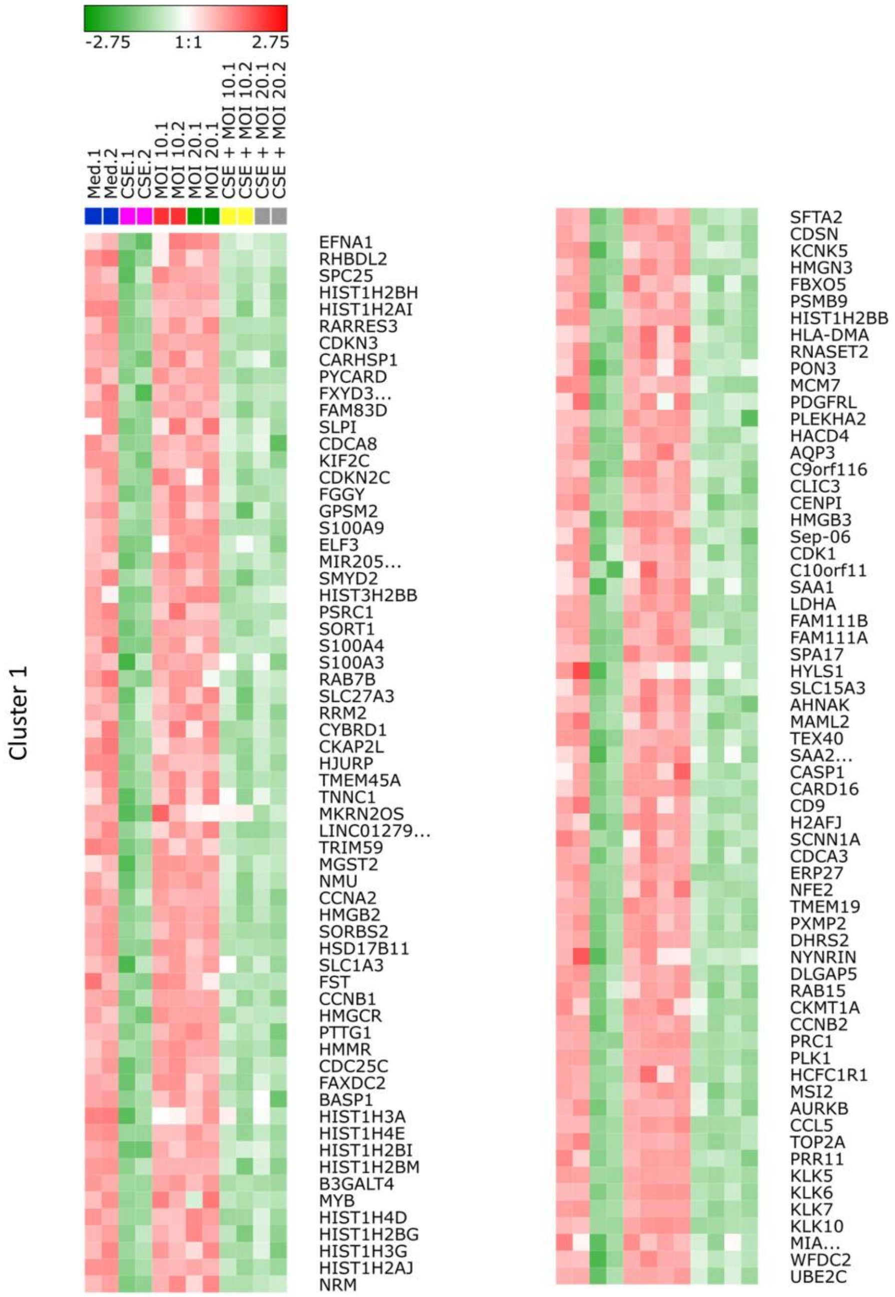
-2.75
1:1
2.75
Med.1
Med.2
CSE.1
CSE.2
MOI 10.1
MOI 10.2
MOI 20.1
MOI 20.1
CSE + MOI 10.1
CSE + MOI 10.2
CSE + MOI 20.1
CSE + MOI 20.2
Cluster 1
EFNA1
RHBDL2
SPC25
HIST1H2BH
HIST1H2AI
RARRES3
CDKN3
CARHSP1
PYCARD
FXYD3...
FAM83D
SLPI
CDCA8
KIF2C
CDKN2C
FGGY
GPSM2
S100A9
ELF3
MIR205...
SMYD2
HIST3H2BB
PSRC1
SORT1
S100A4
S100A3
RAB7B
SLC27A3
RRM2
CYBRD1
CKAP2L
HJURP
TMEM45A
TNNC1
MKRN2OS
LINC01279...
TRIM59
MGST2
NMU
CCNA2
HMGB2
SORBS2
HSD17B11
SLC1A3
FST
CCNB1
HMGCR
PTTG1
HMMR
CDC25C
FAXDC2
BASP1
HIST1H3A
HIST1H4E
HIST1H2BI
HIST1H2BM
B3GALT4
MYB
HIST1H4D
HIST1H2BG
HIST1H3G
HIST1H2AJ
NRM
SFTA2
CDSN
KCNK5
HMGN3
FBXO5
PSMB9
HIST1H2BB
HLA-DMA
RNASET2
PON3
MCM7
PDGFRL
PLEKHA2
HACD4
AQP3
C9orf116
CLIC3
CENPI
HMGB3
Sep-06
CDK1
C10orf11
SAA1
LDHA
FAM111B
FAM111A
SPA17
HYLS1
SLC15A3
AHNAK
MAML2
TEX40
SAA2...
CASP1
CARD16
CD9
H2AFJ
SCNN1A
CDCA3
ERP27
NFE2
TMEM19
PXMP2
DHRS2
NYNRIN
DLGAP5
RAB15
CKMT1A
CCNB2
PRC1
PLK1
HCFC1R1
MSI2
AURKB
CCL5
TOP2A
PRR11
KLK5
KLK6
KLK7
KLK10
MIA...
WFDC2
UBE2C

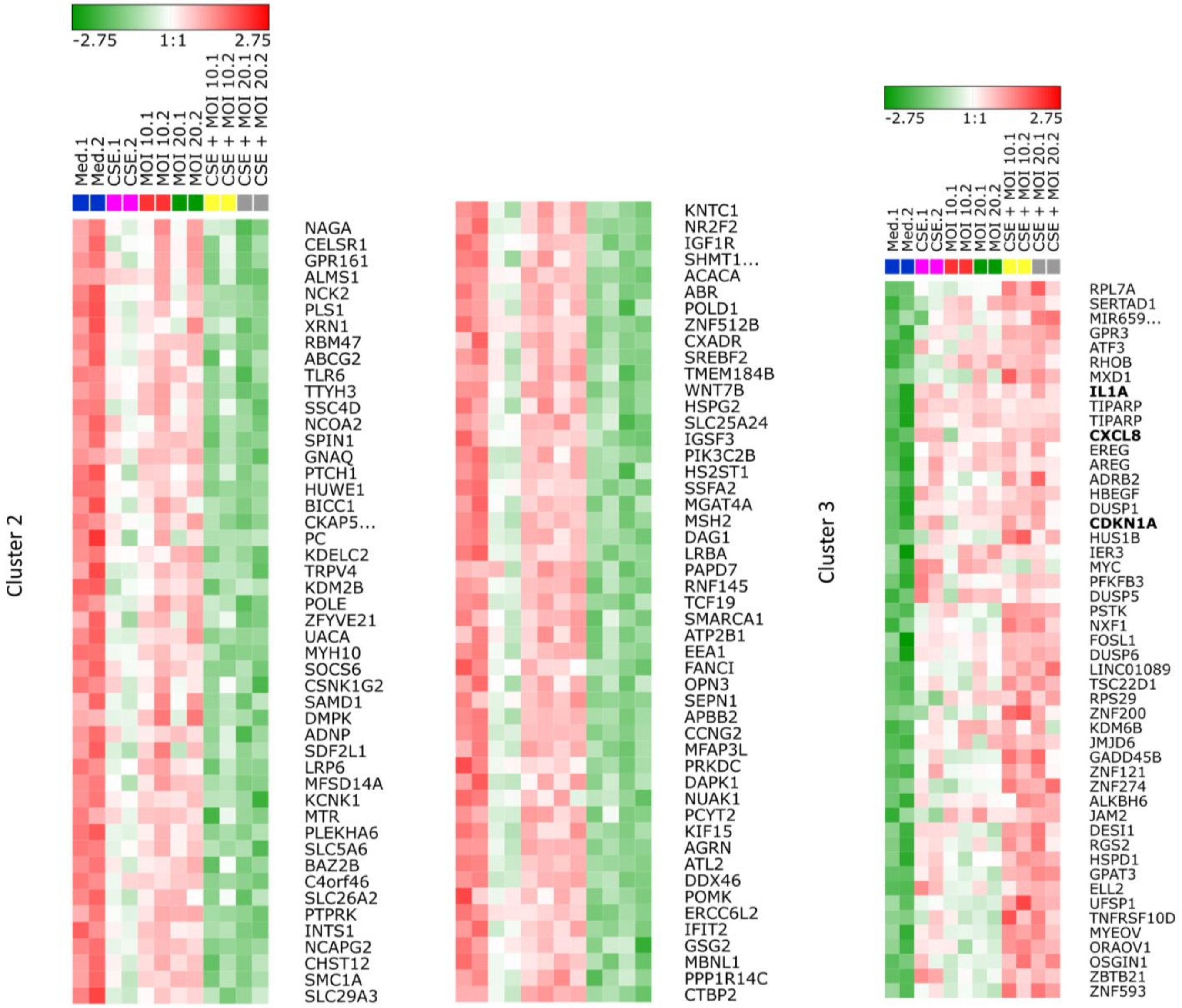
-2.75
1:1
2.75
Med.1
Med.2
CSE.1
CSE.2
MOI 10.1
MOI 10.2
MOI 20.1
MOI 20.2
CSE + MOI 10.1
CSE + MOI 10.2
CSE + MOI 20.1
CSE + MOI 20.2
Cluster 2
NAGA
CELSR1
GPR161
ALMS1
NCK2
PLS1
XRN1
RBM47
ABCG2
TLR6
TTYH3
SSC4D
NCOA2
SPIN1
GNAQ
PTCH1
HUWE1
BICC1
CKAP5...
PC
KDELC2
TRPV4
KDM2B
POLE
ZFYVE21
UACA
MYH10
SOCS6
CSNK1G2
SAMD1
DMPK
ADNP
SDF2L1
LRP6
MFSD14A
KCNK1
MTR
PLEKHA6
SLC5A6
BAZ2B
C4orf46
SLC26A2
PTPRK
INTS1
NCAPG2
CHST12
SMC1A
SLC29A3
KNTC1
NR2F2
IGF1R
SHMT1...
ACACA
ABR
POLD1
ZNF512B
CXADR
SREBF2
TMEM184B
WNT7B
HSPG2
SLC25A24
IGSF3
PIK3C2B
HS2ST1
SSFA2
MGAT4A
MSH2
DAG1
LRBA
PAPD7
RNF145
TCF19
SMARCA1
ATP2B1
EEA1
FANCI
OPN3
SEPN1
APBB2
CCNG2
MFAP3L
PRKDC
DAPK1
NUAK1
PCYT2
KIF15
AGRN
ATL2
DDX46
POMK
ERCC6L2
IFIT2
GSG2
MBNL1
PPP1R14C
CTBP2
Cluster 3
RPL7A
SERTAD1
MIR659...
GPR3
ATF3
RHOB
MXD1
IL1A
TIPARP
TIPARP
CXCL8
EREG
AREG
ADRB2
HBEGF
DUSP1
CDKN1A
HUS1B
IER3
MYC
PFKFB3
DUSP5
PSTK
NXF1
FOSL1
DUSP6
LINC01089
TSC22D1
RPS29
ZNF200
KDM6B
JMJD6
GADD45B
ZNF121
ZNF274
ALKBH6
JAM2
DESI1
RGS2
HSPD1
GPAT3
ELL2
UFSP1
TNFRSF10D
MYEOV
ORAOV1
OSGIN1
ZBTB21
ZNF593

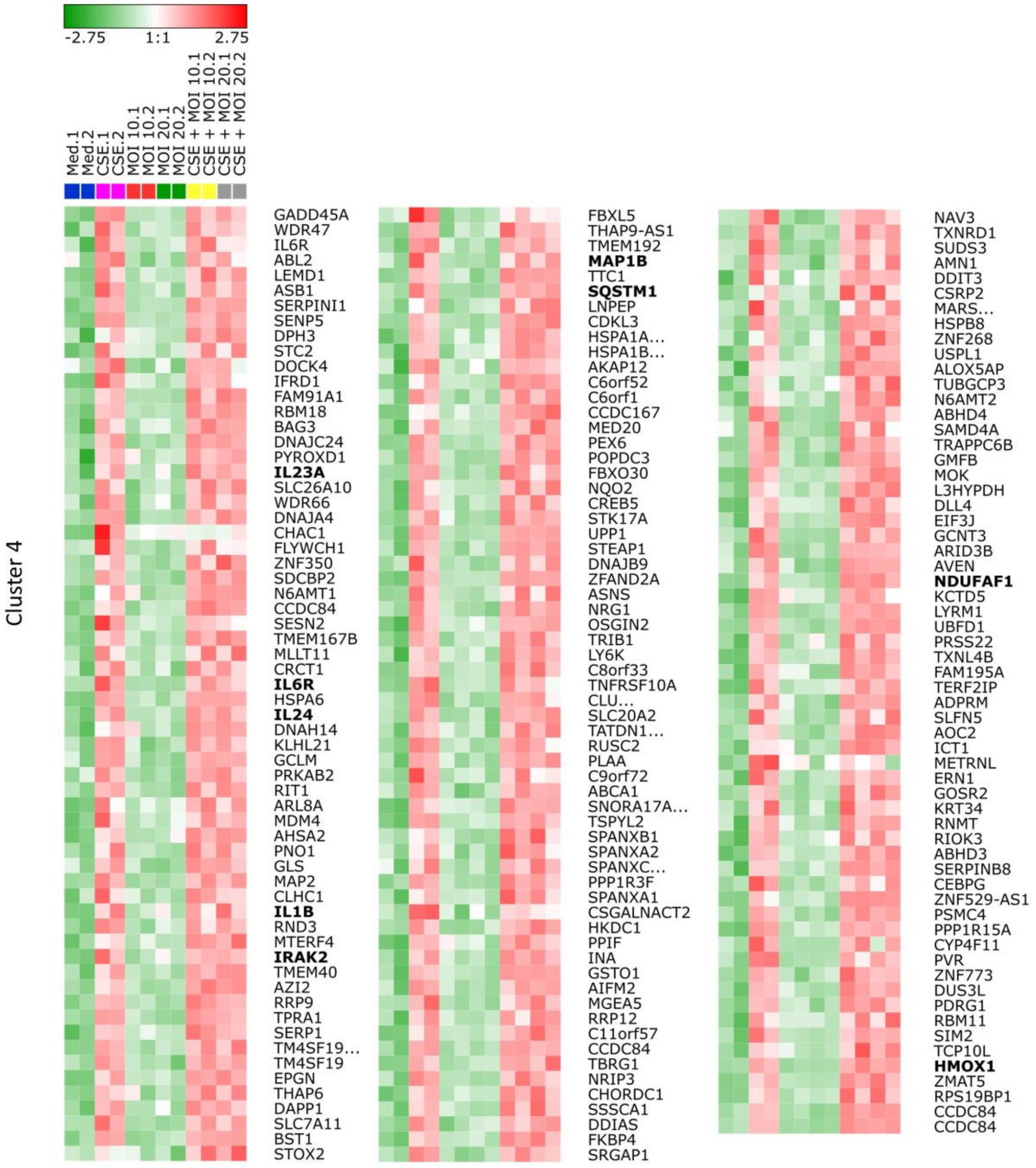
-2.75
1:1
2.75
Med.1
Med.2
CSE.1
CSE.2
MOI 10.1
MOI 10.2
MOI 20.1
MOI 20.2
CSE + MOI 10.1
CSE + MOI 10.2
CSE + MOI 20.1
CSE + MOI 20.2
Cluster 4
GADD45A
WDR47
IL6R
ABL2
LEMD1
ASB1
SERPINI1
SENP5
DPH3
STC2
DOCK4
IFRD1
FAM91A1
RBM18
BAG3
DNAJC24
PYROXD1
IL23A
SLC26A10
WDR66
DNAJA4
CHAC1
FLYWCH1
ZNF350
SDCBP2
N6AMT1
CCDC84
SESN2
TMEM167B
MLLT11
CRCT1
IL6R
HSPA6
IL24
DNAH14
KLHL21
GCLM
PRKAB2
RIT1
ARL8A
MDM4
AHSA2
PNO1
GLS
MAP2
CLHC1
IL1B
RND3
MTERF4
IRAK2
TMEM40
AZI2
RRP9
TPRA1
SERP1
TM4SF19...
TM4SF19
EPGN
THAP6
DAPP1
SLC7A11
BST1
STOX2
FBXL5
THAP9-AS1
TMEM192
MAP1B
TTC1
SQSTM1
LNPEP
CDKL3
HSPA1A...
HSPA1B...
AKAP12
C6orf52
C6orf1
CCDC167
MED20
PEX6
POPDC3
FBXO30
NQO2
CREB5
STK17A
UPP1
STEAP1
DNAJB9
ZFAND2A
ASNS
NRG1
OSGIN2
TRIB1
LY6K
C8orf33
TNFRSF10A
CLU...
SLC20A2
TATDN1...
RUSC2
PLAA
C9orf72
ABCA1
SNORA17A...
TSPYL2
SPANXB1
SPANXA2
SPANXC...
PPP1R3F
SPANXA1
CSGALNACT2
HKDC1
PPIF
INA
GSTO1
AIFM2
MGEA5
RRP12
C11orf57
CCDC84
TBRG1
NRIP3
CHORDC1
SSSCA1
DDIAS
FKBP4
SRGAP1
NAV3
TXNRD1
SUDS3
AMN1
DDIT3
CSRP2
MARS...
HSPB8
ZNF268
USPL1
ALOX5AP
TUBGCP3
N6AMT2
ABHD4
SAMD4A
TRAPPC6B
GMFB
MOK
L3HYPDH
DLL4
EIF3J
GCNT3
ARID3B
AVEN
NDUFAF1
KCTD5
LYRM1
UBFD1
PRSS22
TXNL4B
FAM195A
TERF2IP
ADPRM
SLFN5
AOC2
ICT1
METRNL
ERN1
GOSR2
KRT34
RNMT
RIOK3
ABHD3
SERPINB8
CEBPG
ZNF529-AS1
PSMC4
PPP1R15A
CYP4F11
PVR
ZNF773
DUS3L
PDRG1
RBM11
SIM2
TCP10L
HMOX1
ZMAT5
RPS19BP1
CCDC84
CCDC84

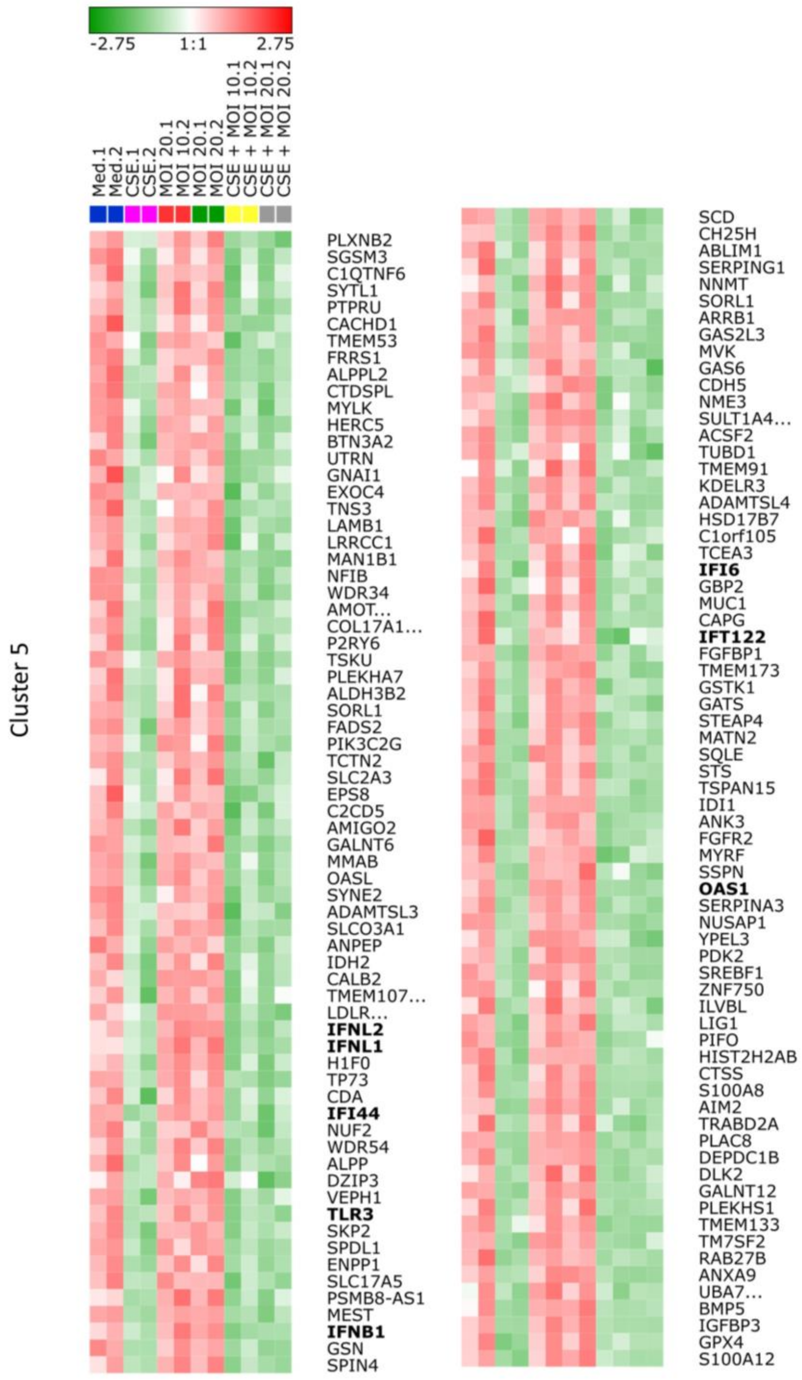

Cluster 5
-2.75
1:1
2.75
Med.1
Med.2
CSE.1
CSE.2
MOI 20.1
MOI 10.2
MOI 20.1
MOI 20.2
CSE + MOI 10.1
CSE + MOI 10.2
CSE + MOI 20.1
CSE + MOI 20.2
PLXNB2
SGSM3
C1QTNF6
SYTL1
PTPRU
CACHD1
TMEM53
FRRS1
ALPPL2
CTDSPL
MYLK
HERC5
BTN3A2
UTRN
GNAI1
EXOC4
TNS3
LAMB1
LRRCC1
MAN1B1
NFIB
WDR34
AMOT...
COL17A1...
P2RY6
TSKU
PLEKHA7
ALDH3B2
SORL1
FADS2
PIK3C2G
TCTN2
SLC2A3
EPS8
C2CD5
AMIGO2
GALNT6
MMAB
OASL
SYNE2
ADAMTSL3
SLCO3A1
ANPEP
IDH2
CALB2
TMEM107...
LDLR...
IFNL2
IFNL1
H1F0
TP73
CDA
IFI44
NUF2
WDR54
ALPP
DZIP3
VEPH1
TLR3
SKP2
SPDL1
ENPP1
SLC17A5
PSMB8-AS1
MEST
IFNB1
GSN
SPIN4
SCD
CH25H
ABLIM1
SERPING1
NNMT
SORL1
ARRB1
GAS2L3
MVK
GAS6
CDH5
NME3
SULT1A4...
ACSF2
TUBD1
TMEM91
KDELR3
ADAMTSL4
HSD17B7
C1orf105
TCEA3
IFI6
GBP2
MUC1
CAPG
IFT122
FGFBP1
TMEM173
GSTK1
GATS
STEAP4
MATN2
SQLE
STS
TSPAN15
IDI1
ANK3
FGFR2
MYRF
SSPN
OAS1
SERPINA3
NUSAP1
YPEL3
PDK2
SREBF1
ZNF750
ILVBL
LIG1
PIFO
HIST2H2AB
CTSS
S100A8
AIM2
TRABD2A
PLAC8
DEPDC1B
DLK2
GALNT12
PLEKHS1
TMEM133
TM7SF2
RAB27B
ANXA9
UBA7...
BMP5
IGFBP3
GPX4
S100A12

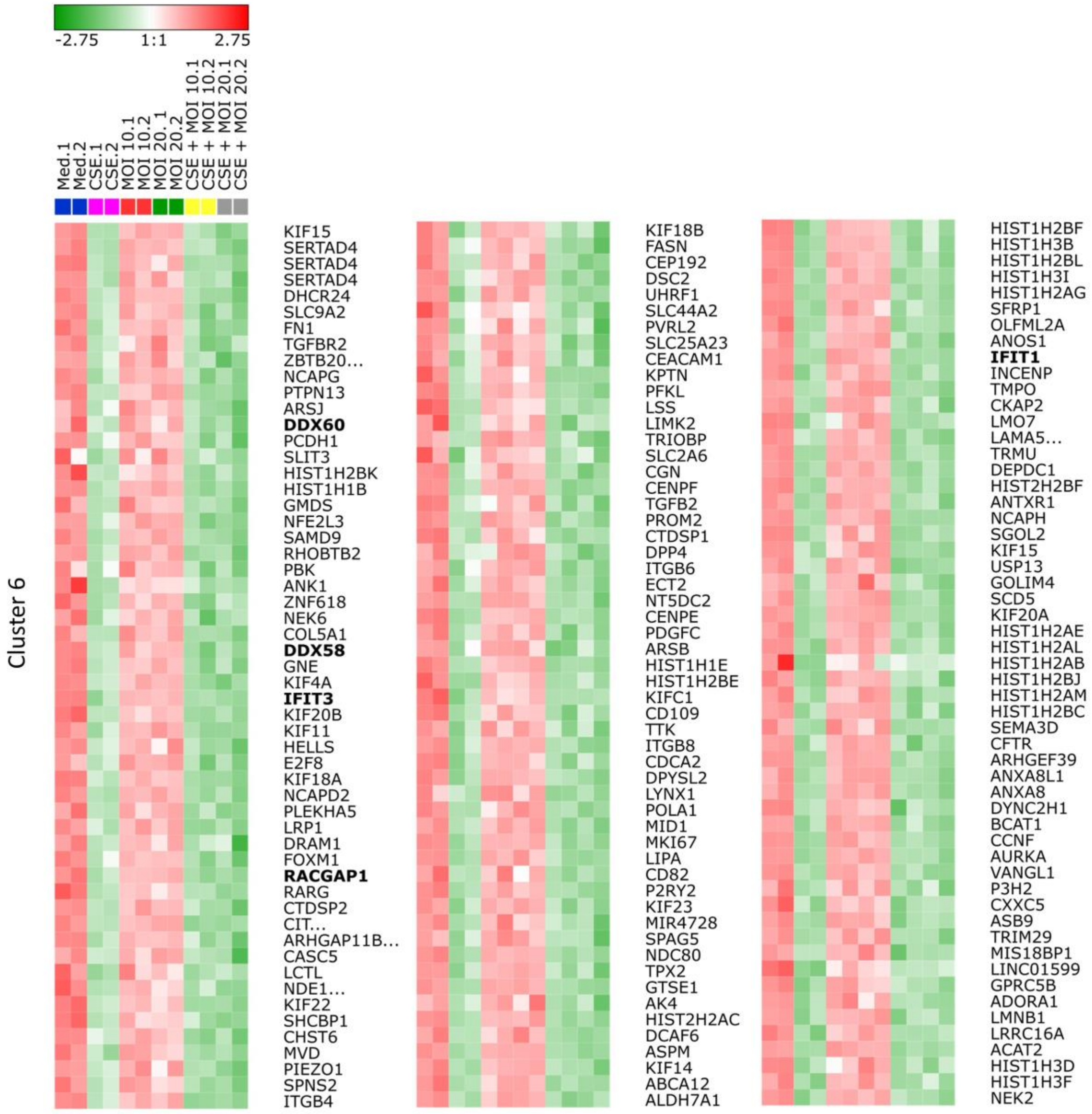
-2.75
1:1
2.75
Med.1
Med.2
CSE.1
CSE.2
MOI 10.1
MOI 10.2
MOI 20.1
MOI 20.2
CSE + MOI 10.1
CSE + MOI 10.2
CSE + MOI 20.1
CSE + MOI 20.2
Cluster 6
KIF15
SERTAD4
SERTAD4
SERTAD4
DHCR24
SLC9A2
FN1
TGFBR2
ZBTB20...
NCAPG
PTPN13
ARSJ
DDX60
PCDH1
SLIT3
HIST1H2BK
HIST1H1B
GMDS
NFE2L3
SAMD9
RHOBTB2
PBK
ANK1
ZNF618
NEK6
COL5A1
DDX58
GNE
KIF4A
IFIT3
KIF20B
KIF11
HELLS
E2F8
KIF18A
NCAPD2
PLEKHA5
LRP1
DRAM1
FOXM1
RACGAP1
RARG
CTDSP2
CIT...
ARHGAP11B...
CASC5
LCTL
NDE1...
KIF22
SHCBP1
CHST6
MVD
PIEZO1
SPNS2
ITGB4
KIF18B
FASN
CEP192
DSC2
UHRF1
SLC44A2
PVRL2
SLC25A23
CEACAM1
KPTN
PFKL
LSS
LIMK2
TRIOBP
SLC2A6
CGN
CENPF
TGFB2
PROM2
CTDSP1
DPP4
ITGB6
ECT2
NT5DC2
CENPE
PDGFC
ARSB
HIST1H1E
HIST1H2BE
KIFC1
CD109
TTK
ITGB8
CDCA2
DPYSL2
LYNX1
POLA1
MID1
MKI67
LIPA
CD82
P2RY2
KIF23
MIR4728
SPAG5
NDC80
TPX2
GTSE1
AK4
HIST2H2AC
DCAF6
ASPM
KIF14
ABCA12
ALDH7A1
HIST1H2BF
HIST1H3B
HIST1H2BL
HIST1H3I
HIST1H2AG
SFRP1
OLFML2A
ANOS1
IFIT1
INCENP
TMPO
CKAP2
LMO7
LAMA5...
TRMU
DEPDC1
HIST2H2BF
ANTXR1
NCAPH
SGOL2
KIF15
USP13
GOLIM4
SCD5
KIF20A
HIST1H2AE
HIST1H2AL
HIST1H2AB
HIST1H2BJ
HIST1H2AM
HIST1H2BC
SEMA3D
CFTR
ARHGEF39
ANXA8L1
ANXA8
DYNC2H1
BCAT1
CCNF
AURKA
VANGL1
P3H2
CXXC5
ASB9
TRIM29
MIS18BP1
LINC01599
GPRC5B
ADORA1
LMNB1
LRRC16A
ACAT2
HIST1H3D
HIST1H3F
NEK2

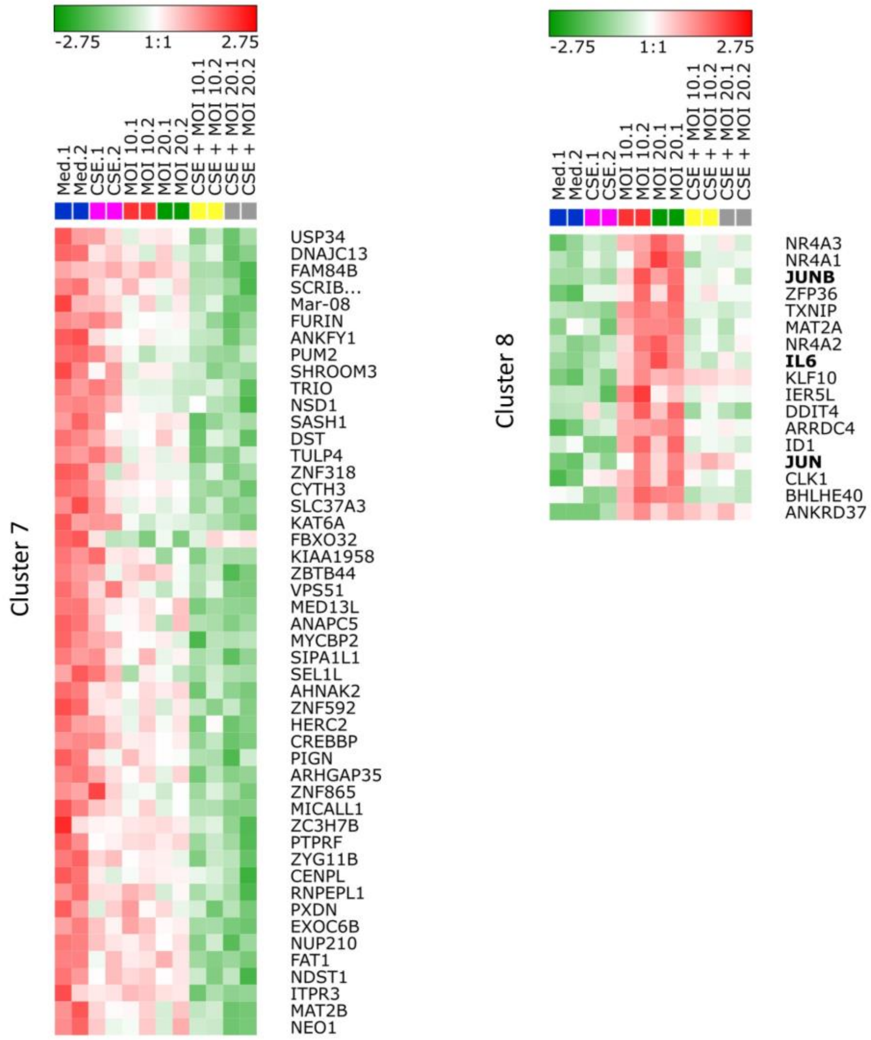

Figure S5: Microarray analysis of gene expression upon CSE stimulation and *Streptococcus pneumoniae* infection. 16HBE cells were stimulated with medium (Med) or cigarette smoke extract (CSE) for 28 h, CSE for 24 h followed by medium 4 h (CSE cessation), *Streptococcus pneumoniae* (*Sp*) multiplicities of infection (MOI) of 10 and 20 for 4 h, CSE for 24 h followed by *Sp* MOI 10 and 20 for 4 h. Total RNA was isolated and investigated by using human Clariom S microarray. Differentially expressed transcripts were determined by comparing each condition with medium control (fold change ≥ 3-fold, false discovery rate < 0.05). Log_2 signal intensities data of the transcripts were z-score transformed and k-means clustered (k = 8), and transcripts in resulting clusters were sorted by maximal absolute z-score (row labels: gene symbols). Data represent color-coded z-scores. Details on bold gene symbols are mentioned in the text.

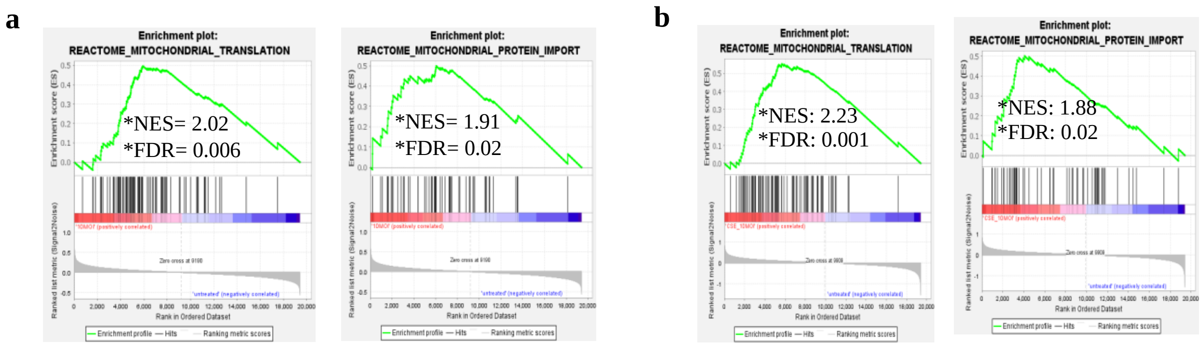

Sp MOI 10 CSE + Sp MOI 10

Figure S6: Expression of genes regulating mitochondrial function in 16HBE cells upon *Streptococcus pneumoniae* infection with and without CSE pre-exposure. Gene set enrichment analysis (GSEA) for mitochondrial ribosomal translation and mitochondrial import Reactome genes upon **a)** infecting 16HBE cells with *Streptococcus pneumoniae* (*Sp*) multiplicity of infection (MOI) 10 for 4 h and **b)** incubation with cigarette smoke extract (CSE) for 24 h followed by *Sp* infection with MOI 10 for 4 h compared to the untreated medium (ranked by fold change, FDR < 0.1). The data were collected from two independent experiments.

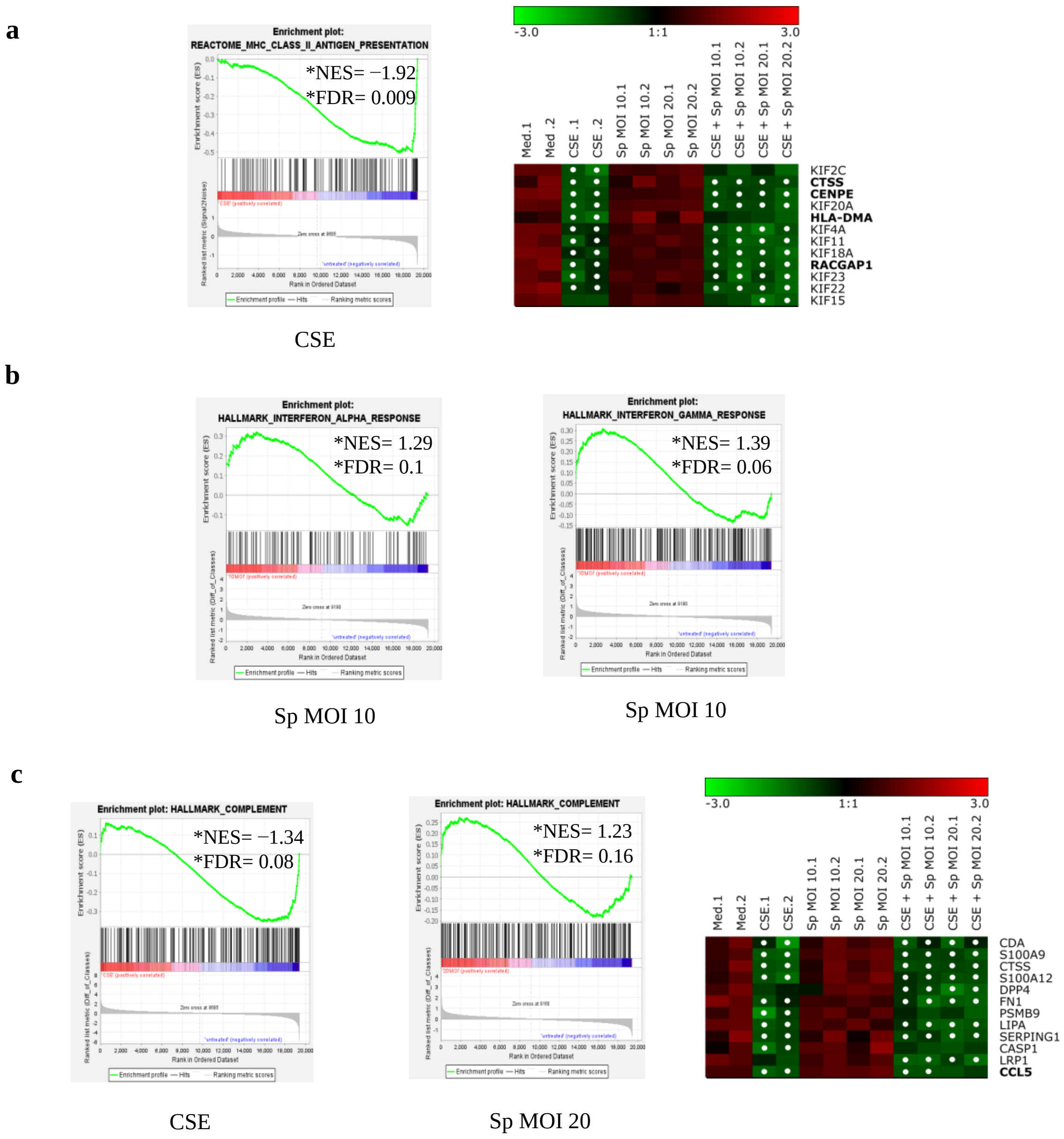

Figure S7: Expression of genes regulating innate immune responses upon cigarette smoke extract stimulation and *Streptococcus pneumoniae* infection. **a)** Gene set enrichment analysis (GSEA) for cell surface major histocompatibility complex II (MHCII) in Reactome gene set upon cigarette smoke extract (CSE) stimulation compared to the medium (Med) control. Normalized $\log_2$ signal intensities of core-enriched genes were z-score transformed, color-coded and plotted as heatmap (row labels: gene symbols). Details on bold gene symbols are mentioned in the text. Significance of differential expression with fold change cutoff $|FC| > 3$ in reference to Med control was calculated by ANOVA ($p<0.05$). Whitepoints in heatmap plots indicate $|FC| > 3$ with $p < 0.05$. Green/black/red color bars represent z-scores. **b)** GSEA for interferon α and γ hallmarks upon infection of 16HBE cells with multiplicity of infection (MOI) 10 of *Streptococcus pneumoniae* (*Sp*). **c)** GSEA for complement hallmarks upon stimulation of 16HBE cells with CSE for 28 h and *Sp* MOI 20 for 4 h compared to the untreated medium as well as regulated genes in this hallmark calculated by ANOVA ($p < 0.05$). The data were collected from two independent experiments. *NES: Normalized enrichment score, FDR: false discovery rate.

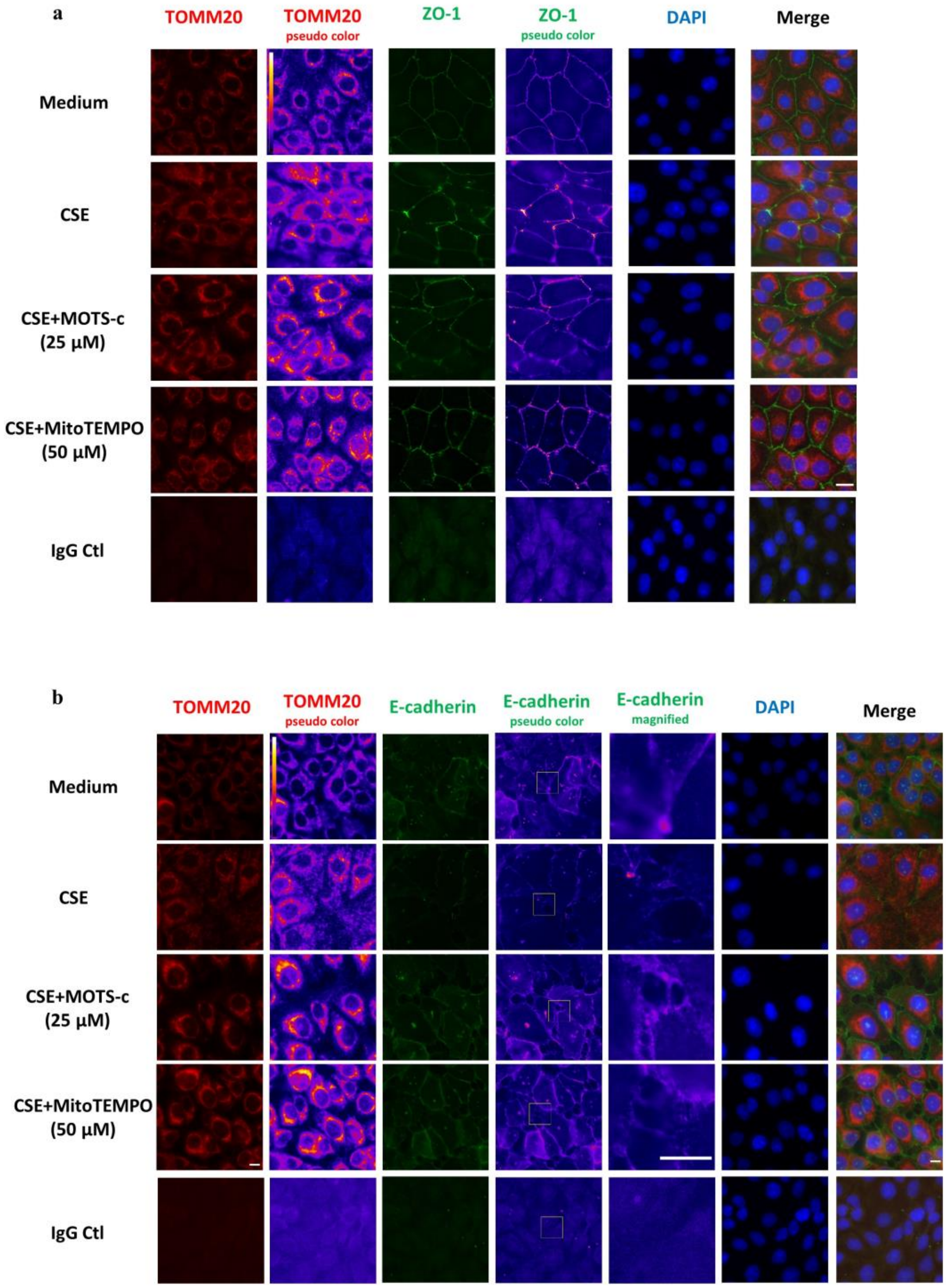

Figure S8: Airway epithelial barrier disruption induced by cigarette smoke extract is reversible with mitochondrial-targeted compounds. 16HBE cells incubated for 28 h with medium (control) or cigarette smoke extract (CSE) for 28 h, or for 24 h with CSE followed by incubation for further 4 h with either mitochondrial-targeted compounds MOTS-c (25 µM) or MitoTEMPO (50 µM). a) Immunofluorescence imaging of treated cells co-stained with antibodies against translocase of the outer mitochondrial membrane complex subunit 20 (TOMM20) in red, tight junction protein zonula occludens (ZO-1) in green or the nucleus with 4',6-diamidino-2-phenylindole (DAPI) for nucleus staining (blue). b) Treated cells were (co-)stained with anti-TOMM20-antibody (red) and anti-E-cadherin-antibody (green) or with

nuclear staining dye DAPI (blue). False color-coded image columns shown here to depict differences in intensities. To visualize the barrier disruption, magnified regions of interest are indicated in the fourth column and shown in column five. Merged images have been gamma corrected to visualize weak signals without losing the highlights. The scale bar is equivalent to 20 µm (a) and 10 µM (b).

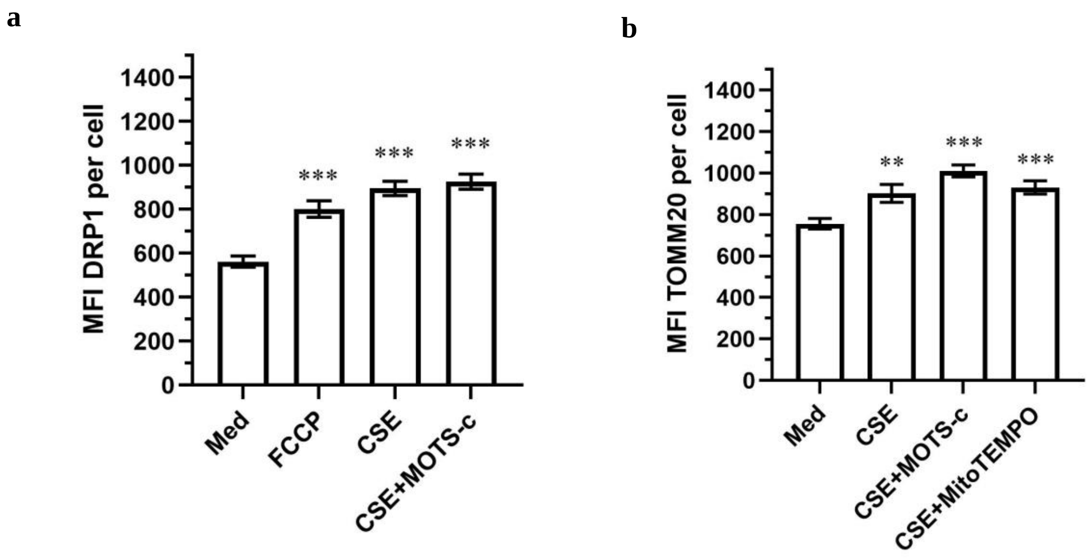

Figure S9: Quantification of signal intensities of immunofluorescence staining in 16HBE cells. The cells were stained with antibodies against **a)** dynamin-related protein 1 (DRP1) and **b)** translocase of the outer mitochondrial membrane complex subunit 20 (TOMM20). Mean fluorescence intensity (MFI) was calculated for each cell in the corresponding immunofluorescence images by Fiji software. The statistically significant differences with the medium (Med) were determined by unpaired t-test and using Welch's post-hoc test (**p<0.001, ***p<0.0001).
